# Investigating causal associations among gut microbiota, metabolites, and psoriatic arthritis: a Mendelian randomization study

**DOI:** 10.3389/fmicb.2024.1287637

**Published:** 2024-02-15

**Authors:** Xiao Xu, Lin-yun Wu, Shu-yun Wang, Min Yan, Yuan-Hong Wang, Li Li, Zhi-ling Sun, Ji-Xiang Zhao

**Affiliations:** ^1^Department of Nursing, Nantong Health College of Jiangsu Province, Nantong, China; ^2^School of Nursing, Zhejiang Chinese Medical University, Hangzhou, China; ^3^Academic Affair Office, Nantong Vocational University, Nantong, China; ^4^Department of Epidemiology, School of Public Health, Changzhou University, Changzhou, China; ^5^Faculty of Health and Welfare, Satakunta University of Applied Sciences, Pori, Finland; ^6^Department of Rheumatology, The Affiliated Hospital of Nanjing University of Chinese Medicine, Nanjing, China; ^7^Department of Epidemiology, School of Public Health, Nanjing University of Chinese Medicine, Nanjing, China; ^8^Department of Nursing, The Affiliated Suzhou Hospital of Nanjing Medical University, Suzhou, China

**Keywords:** data-driven approach, gut microbiota, Mendelian randomization, metabolites, psoriatic arthritis

## Abstract

**Background:**

Currently, there has been observed a significant alteration in the composition of the gut microbiome (GM) and serum metabolites in patients with psoriatic arthritis (PsA) compared to healthy individuals. However, previous observational studies have shown inconsistent results regarding the alteration of gut microbiota/metabolites. In order to shed light on this matter, we utilized Mendelian randomization to determine the causal effect of GM/metabolites on PsA.

**Methods:**

We retrieved summary-level data of GM taxa/metabolites and PsA from publicly available GWAS statistics. Causal relationships between GM/metabolites and PsA were determined using a two-sample MR analysis, with the IVW approach serving as the primary analysis method. To ensure the robustness of our findings, we conducted sensitivity analyses, multivariable MR analysis (MVMR), and additional analysis including replication verification analysis, LDSC regression, and Steiger test analysis. Furthermore, we investigated reverse causality through a reverse MR analysis. Finally, we conducted an analysis of expression quantitative trait loci (eQTLs) involved in the metabolic pathway to explore potential molecular mechanisms of metabolism.

**Results:**

Our findings reveal that eight GM taxa and twenty-three serum metabolites are causally related to PsA (*P* < 0.05). Notably, a higher relative abundance of Family *Rikenellaceae* (OR_*IVW*_: 0.622, 95% CI: 0.438–0.883, FDR = 0.045) and elevated serum levels of X-11538 (OR_*IVW*_: 0.442, 95% CI: 0.250–0.781, FDR = 0.046) maintain significant causal associations with a reduced risk of PsA, even after adjusting for multiple testing correction and conducting MVMR analysis. These findings suggest that Family *Rikenellaceae* and X-11538 may have protective effects against PsA. Our sensitivity analysis and additional analysis revealed no significant horizontal pleiotropy, reverse causality, or heterogeneity. The functional enrichment analysis revealed that the eQTLs examined were primarily associated with glycerolipid metabolism and the expression of key metabolic factors influenced by bacterial infections (*Vibrio cholerae* and *Helicobacter pylori*) as well as the mTOR signaling pathway.

**Conclusion:**

In conclusion, our study demonstrates that Family *Rikenellacea*e and X-11538 exhibit a strong and negative causal relationship with PsA. These particular GM taxa and metabolites have the potential to serve as innovative biomarkers, offering valuable insights into the treatment and prevention of PsA. Moreover, bacterial infections and mTOR-mediated activation of metabolic factors may play an important role in this process.

## 1 Introduction

Psoriatic arthritis (PsA) is a long-lasting inflammatory disorder characterized by both skin manifestations of psoriasis, such as psoriasis-like skin lesions, nail changes, and dactylitis, as well as inflammation in the musculoskeletal system, manifested as peripheral arthritis, enthesitis, axial involvement, and more (FitzGerald et al., 2021). A comprehensive analysis conducted by Scotti et al. (2018) revealed that PsA, defined using Classification Criteria for Psoriatic Arthritis (CASPAR) criteria, affects approximately 23.8% of individuals who have psoriasis. This substantial prevalence of PsA among psoriasis patients not only causes significant disability but also hinders daily functioning, working performance, and productivity (Coates et al., 2022). Furthermore, the presence of comorbidities, such as cardiovascular diseases and metabolic syndrome, greatly influences the life expectancy, overall health, and quality of life of those affected (Gupta et al., 2021). The chronic pain and physical limitations associated with PsA can also have a profound impact on mental wellbeing, contributing to elevated rates of psychological distress (Kılıç et al., 2023).

The precise development of PsA remains not fully comprehended, however, it is thought to involve a complicated interplay of immunogenetic factors (such as: HLA-B27, HLA-Cw6, IL23R, etc.), as well as environmental factors (like stress, infection, physical trauma, etc.) (Ritchlin, 2007). At present, advanced OMIC technologies have been implemented to investigate the underlying pathophysiological mechanisms of PsA (Gurke et al., 2022). There is a growing body of evidence indicating that changes in the gut microbiota (GM) and metabolic molecules have a substantial impact on the development and progression of PsA (Olejniczak-Staruch et al., 2021; Radulska et al., 2023). Specifically, dysbiosis causes the imbalance between the various subtypes of CD4^+^ T cells that leads to chronic inflammation in the joints, resulting in a range of clinical symptoms such as arthritis, tendonitis, sacroiliitis, and more (Schatteman et al., 1995). Importantly, Th17 cells are known to produce cytokines such as IL-17A and IL-17F, which can cause chronic joint inflammation in patients with PsA (Chimenti et al., 2013). Furthermore, recent studies have highlighted the association between the composition of the gut microbiota and chronic inflammatory conditions, such as PsA, partly attributed to alterations in the concentration of metabolites produced by gut microbes.

Several observational studies have explored the composition of the gut microbiota and metabolites in patients with psoriasis, but research on psoriatic arthritis (PsA) remains limited (Myers et al., 2019). From the metabolomic perspective, Ambrożewicz et al. (2018) conducted an observational study comprising 34 patients diagnosed with psoriatic arthritis at the Medical University of Bialystok. The lipid profile was analyzed using Hydrophilic interaction liquid chromatography (HILIC-LC)-MS, revealing a significant reduction in plasma levels of fatty acids, including Phospholipid LA (18:2), LA (18:3), free AA (20:4), and DHA (22:6), in PsA patients compared to healthy individuals. However, another cross-sectional study performed by Looby et al. (2021) presented contradictory results, indicating that the serum levels of long-chain fatty acids involved in lipid metabolism were elevated instead This contradictory evidence underscores the inconsistency in the association between metabolic levels and PsA as identified in previous research.

From the GM perspective, to date, only two research studies have investigated the relationship between gut microbiota and the likelihood of PsA. Most of the existing research on this subject is part of the broader field of spondyloarthritis (Eppinga et al., 2014). Scher et al. (2015) conducted a study comparing the composition of the gut microbiome in individuals with recent-onset PsA, as well as healthy controls. The findings revealed a marked reduction in overall diversity of fecal microbiomes in individuals with PsA compared to those of healthy individuals. Furthermore, there was a noticeable decrease in the presence of certain genera levels, including *Akkermansia*, *Ruminococcus*, *Pseudobutyrivibrio*, *Parabacteroides*, *Alistipes*, and *Coprococcus*, in individuals with PsA compared to the healthy cohort. Furthermore, Manasson et al. (2020) recruited a cohort of fifteen SpA patients from the NYU clinics before commencing treatment with biologics. Through the utilization of 16s rRNA (V4) Illumina Miseq technology, they noted a marked rise in levels of *Clostridiales* and *Erysipelotrichaceae*, alongside a decrease in levels of *Bacteroidales* at the order level compared to the control group of healthy subjects. Hence, additional studies are imperative to bolster the support for the relationship between gut microbiota and PsA, and to address the discrepancies in findings regarding the correlation between serum metabolites and PsA in existing research.

Furthermore, previous observational studies conducted with small sample sizes have inherent limitations that can impact the credibility and interpretation of their findings. One notable limitation pertains to the existence of confounding variables. Various confounding variables, including dietary habits, lifestyle factors, and pharmacotherapy, have the potential to influence both the composition of GM/metabolites as well as the likelihood of developing PsA (Vujkovic-Cvijin et al., 2020). Another limitation found in observational studies is the issue of reverse causality. The intricate interplay among gut microbiota, metabolites, and PsA complexities makes it difficult to determine the direction of causality and underlying mechanisms involved.

Taking these limitations into consideration, Walter et al. (2020) propose the use of Mendelian randomization (MR) as a state-of-the-art statistical method for determining the causal role of GM/metabolites in disease progression. This innovative method employs genetic variants as IVs to assess causality. By taking advantage of the random assortment of genetic variants during meiosis, MR enables causal inference similar to that of a randomized controlled trial, thus reducing the impact of confounding factors and the bias of reverse causation that often arise in observational studies (Lawlor et al., 2008).

Hence, our research initially utilized a Mendelian randomization methodology to identify the causal connections among the composition of gut microbiota, production of metabolites, and PsA. The findings from our study have the potential to bring about groundbreaking insights into the underlying mechanisms of PsA.

## 2 Materials and methods

### 2.1 Study design and ethics approval statement

**FIGURE 1:**
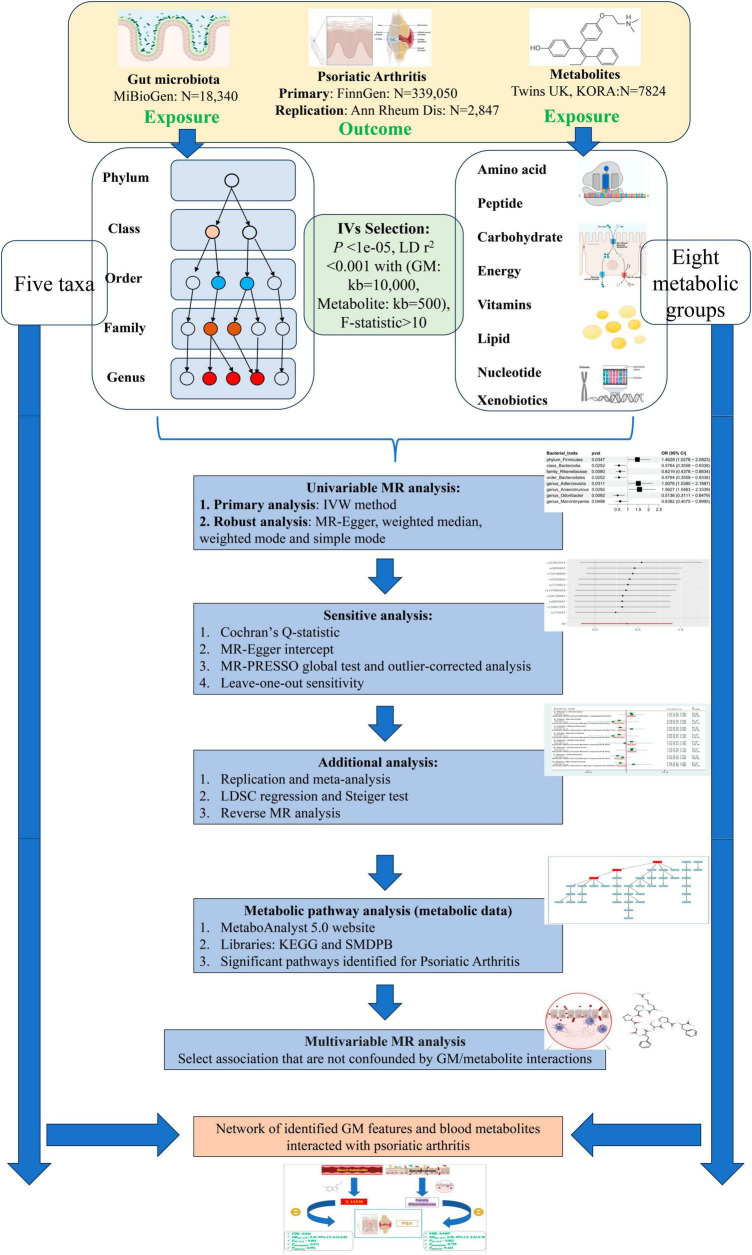
Flowchart of overview of Mendelian randomization (MR) analysis.

Figure 1 Depicts the flowchart outlining the MR analysis conducted to evaluate the causal effects of GM taxa/metabolites on PsA. Additionally, it illustrates the network analysis performed to examine the interaction between identified GM taxa and metabolites with PsA. The study design adhered to the MR_STROBE guidance (Skrivankova et al., 2021; Supplementary Table 1). Summary-level data of GM taxa/metabolites and PsA were retrieved from publicly available GWAS statistics. SNPs significantly associated with GM taxa and metabolites were extracted as instrumental variables (IVs). Furthermore, each original GWAS study involved in this MR research obtained ethical approval and informed consent from their respective institutions.

### 2.2 Data sources

Detailed information regarding the dataset used is provided in Table 1.

**TABLE 1:** Sources and description of summary statistics used in the study.

Phenotypes	Consortium PMID	Sample size	Ethnicity	Sex	Age	Eligibility criteria	Other comorbidities
Psoriatic arthritis	FinnGen Biobank	4, 278 cases 39, 029 controls	Eur	Female in cases (*N* = 2390) Male in cases (*N* = 1888)	Median age: 51.78 years	Diagnostic criteria according to ICD10–M07.1, M07.1 L40.5, M07.2, M07.2 L40.5, M07.3, M07.3 L40.5	Essential Hypertension; Dermatitis; Acute and subacute iridocyclitis Dry eye syndrome; Type 2 diabetes mellitus; Asthma; Fracture of rib
GM	MiBioGen 33462485	18, 340 cases	Multiple (85% Eur)	Female in cases (*N* = 10,232) Male in cases (*N* = 8,108)	Median age: 46.01 years	Healthy individuals	N/A
Metabolite	TwinsUK and KORA cohorts 24816252	7, 824 cases	Eur	Female in cases (*N* = 6,533) Male in cases (*N* = 1,291)	Median age: 57.11 years	Healthy individuals	N/A

#### 2.2.1 Gut microbiota GWAS data source

The summary statistics for the gut microbiota were obtained from the international consortium MiBioGen (website)^1^ (Kurilshikov et al., 2021). The MiBioGen consortium coordinated 16S rRNA gene sequencing profiles and genotyping data from 18,340 individuals across 24 cohorts in the USA, Canada, Israel, South Korea, Germany, Denmark, Netherlands, Belgium, Sweden, and the UK. This dataset involved a large-scale, multi-ethnic, genome-wide meta-analysis of the associations between autosomal human genetic variants and the gut microbiome, representing the most comprehensive examination of the genetic factors affecting the human gut microbiota to date. Specifically, quality control measures were performed by Kurilshikov et al. (2021) with a requirement for a pointwise imputation quality control>0.4 for inclusion in the analysis. Taxa with a prevalence lower than 20% were discarded. The microbiome GWAS was adjusted for age, sex, technical covariates, and genetic principal components. The cutoff thresholds included a minor allele frequency >0.05 and SNP-wise call filtering >0.95. Kurilshikov et al. (2021) reported significant loci of host genetic variation in relation to microbial taxa. After excluding 15 unknown taxa, there were 196 known bacterial taxa, including 9 phyla, 16 classes, 20 orders, 32 families, and 119 genera.

#### 2.2.2 Metabolite GWAS data source

The most comprehensive genetic data for human blood metabolites were accessed through the Human Metabolome Genome-Wide Association Study (HMGWAS) server (website)^2^ (Shin et al., 2014). This dataset represents the most extensive investigation to date on the genetic impact on human serum metabolism (Shin et al., 2014). It comprises metabolic data from 7,824 adult participants of European ancestry across two separate European cohort studies (6,056 participants from the TwinsUK and 1,768 individuals from the KORA F4 study in Germany).

Briefly, the TwinsUK cohort is an adult twin British registry composed mostly of women. These twins were recruited from the general UK population through national media campaigns and have shown similar disease-related and lifestyle characteristics to population-based singletons in the same age group (Moayyeri et al., 2013). The Cooperative Health Research in the Region of Augsburg (KORA) F4 study is a series of independent population-based epidemiological surveys and follow-up studies of participants living in the region of Augsburg, Southern Germany (Wichmann et al., 2005).

Serum metabolites from KORA and TwinsUK participants were quantified at Metabolon Inc. (Durham, NC, USA) using a non-targeted metabolomics approach, specifically gas and liquid chromatography coupled to mass spectrometry (GC/MS and LC/MS, respectively). Details of the methods applied for quantification and identification of metabolites were reported previously (Evans et al., 2009; Dehaven et al., 2010). According to the KEGG database, the established metabolites are categorized into eight major metabolic groups: amino acids, carbohydrates, cofactors and vitamins, energy, lipids, nucleotides, peptides, and xenobiotics (Kanehisa and Goto, 2000).

#### 2.2.3 Outcome data of PsA

In accordance with previously published MR studies (Cao et al., 2023; Wang et al., 2023d), the present study primarily utilized the largest GWAS summary data on PsA, which was freely accessed from the FinnGen Biobank (Kurki et al., 2023). The FinnGen Biobank study is a nationwide collection of genotyped samples from Finnish individuals, comprising both genetic and lifelong health record data from all participants. This enables the investigation of potentially shared and distinct genetic landscapes associated with PsA. It provides an opportunity for both GWAS and cross-disease analyses to better understand potential shared genetic contributors. As an isolated population, Finns have a more homogeneous genetic makeup than others. This dataset comprises 8,075 cases and 339,050 controls in relation to PsA outcomes. PsA patients are primarily diagnosed based on the ICD-10 codes, with a small proportion diagnosed using ICD-9 (6960) and ICD-8 (6960) codes.

### 2.3 The selection of optimal instrumental variables

The selection of optimal IVs should comply with the following three key assumptions of Mendelian randomization (MR): (i) the IVs selected for analysis should be robustly associated with bacterial taxa and metabolites; (ii) the IVs should be independent of potential confounders; and (iii) the IVs should only affect the outcome of PsA through the mediation of bacterial taxa or metabolites, indicating the absence of horizontal pleiotropy (Burgess et al., 2016).

First, in order to obtain a more comprehensive result, we chose independent SNPs that were strongly associated with bacterial taxa with a *P*-value less than 1 × 10^–5^, instead of the conventional threshold of *P* < 5 × 10^–8^. This adjustment was made in consideration of previous studies published in the field (Jia et al., 2019; Sanna et al., 2019; Lv et al., 2021; Ni et al., 2021) . Similarly, IVs for metabolites were obtained using a significance level of *P* < 1 × 10^–5^, following previous research (Yun et al., 2023). Second, we applied a clumping process with *R*^2^ = 0.001 and a genetic distance of 10,000 kb to minimize the impact of linkage disequilibrium (LD) in the European ancestral individuals from the 1000 Genomes Project using the R package (Abecasis et al., 2010). Third, SNPs with minor allele frequency (MAF) of less than 0.01 were removed. Fourth, proxy single nucleotide polymorphisms (SNPs) with a linkage disequilibrium (LD) threshold of *R*^2^ > 0.8 were used to substitute for GM/metabolite-related SNPs that were not available in the PsA GWAS summary data (Meesters et al., 2012). Fifth, in order to ensure that the effect alleles belong to the same allele, we harmonized the exposure (GM/metabolites) and outcome (PsA) datasets to eliminate ambiguous SNPs with non-concordant alleles, SNPs that are palindromic with intermediate allele frequencies, and duplicate SNPs. Sixth, the F-statistic [formula: (*R*^2^/(1-*R*^2^)) × ((N–K-1)/K)] was calculated for each exposure to assess the strength of the selected SNPs. SNPs with an F-statistic less than 10 were eliminated to avoid potential bias from weak IVs (Yengo et al., 2018). Lastly, to mitigate potential biases resulting from inadequate instrumental variables (IVs) and to ensure sufficient statistical power for valid causal inference, we removed both GMs and metabolites with less than 0.5% variance explained by genetic variants (Jia et al., 2023; Wang et al., 2023c). Furthermore, the relative bias of the IV estimate from the 2SLS method, defined as the bias of the IV estimate divided by the bias of the observational estimate, is approximately equal to 1/E(*F*), where E(*F*) represents the expected value of the F statistic. This approximation is only valid when the number of IVs is at least 3 (Staiger and Stock, 1994). Additionally, some MR sensitivity analysis required at least 3 SNPs related to exposure as the genetic instrument (Hemani et al., 2018; Pan et al., 2019). Therefore, both the GMs and metabolites associated with less than 3 SNPs were excluded in this MR study to meet the minimum requirement of the number of SNPs for F statistic calculation and MR sensitivity analysis (Hemani et al., 2018). Furthermore, we calculated the statistical power (>80%) of potential eligible candidate GMs/metabolites at a significance level of 0.05 using an online mRnd power calculator^3^ based on the method described by Burgess (2014).

### 2.4 Statistical analysis

The IVW high-efficiency approach was applied as the primary analysis to determine the causal link between gut microbiome composition/metabolites and PsA (Yang et al., 2012). The IVW method was primarily employed for fundamental causal estimates, which would provide the most precise results when all selected SNPs were valid IVs. The IVW method calculates a weighted average of Wald ratio estimates (Burgess et al., 2013). Complementary approaches including MR-Egger, weighted median, weighted mode, and simple mode were also utilized to evaluate the robustness of results (Bowden et al., 2015; Bowden et al., 2016) . Under the assumption of Instrument Strength Independent of Direct Effect (InSIDE), the MR-Egger regression executes a weighted linear regression and yields a consistent causal estimate, even though the genetic IVs are all invalid (Bowden et al., 2015). However, it exhibits low precision and is susceptible to outlying genetic variants. The Weighted Median regression method, which does not demand the InSIDE hypothesis, calculates a weighted median of the Wald ratio estimates and is robust to horizontal pleiotropic bias (Bowden et al., 2016). It is confirmed that the Weighted Median method has some advantages over the MR-Egger regression, as it provides lower type I error and higher causal estimate power. The Weighted Mode method estimates the causal effect of the subset with the largest number of SNPs by clustering the SNPs into subsets resting on the resemblance of causal effects (Hartwig et al., 2017). The simple mode is a model-based estimation method that provides robustness for pleiotropy, although it is not as powerful as IVW (Hartwig et al., 2017). Inconsistent results between the main method and complementary methodologies were resolved by giving precedence to the IVW method.

We assessed horizontal pleiotropy using the MR-Egger intercept test, which performs weighted linear regression with the intercept unconstrained (Bowden et al., 2015). The intercept represents the average pleiotropic effect across the genetic variants (the average direct effect of a variant with the outcome). If the intercept differed from zero (MR-Egger intercept *P* < 0.05), there was evidence of horizontal pleiotropy. Moreover, we applied the MR-Pleiotropy Residual Sum and Outlier methods (MR-PRESSO) method for detecting overall horizontal pleiotropy (i.e., the global test), identifying specific outliers (i.e., the outlier test), and recalculating effect estimates after outlier removal (Verbanck et al., 2018). MR-PRESSO method is less biassed and more accurate than IVW and MR-Egger when the percentage of horizontal pleiotropy is less than 10% (Ong and MacGregor, 2019). To find heterogeneity, we employed the IVW approach and MR-Egger regression, the heterogeneities were quantified by Cochran’s Q statistic (smaller *P*-values indicate higher heterogeneity and higher potential for directional pleiotropy) (Hemani et al., 2018). The random-effect model of IVW is utilized in cases of heterogeneity (*P* < 0.05), while the fixed-effect IVW model is applied in its absence (*P* > 0.05).

In confounding analysis, we evaluated each SNP and its proxy in the PhenoScanner GWAS database (Kamat et al., 2019)^4^ to carefully assess their associations with potential confounding traits (including smoking, alcohol consumption, hypertension, dyslipidemia, obesity and type 2 diabetes mellitus) among GM/metabolites and the risk of PsA, which can identify potential pleiotropic effects (Näslund-Koch et al., 2023; Zhang Z. Y. et al., 2023). Once the SNPs were associated with these potential confounders at the threshold of *P* < 1 × 10^–5^, IVW was replicated after dropping these SNPs to eliminate the risk of horizontal pleiotropy in our MR findings and validate the robustness of the results.

Furthermore, multivariable MR (MVMR) analysis, extended from MR approach, is capable of capturing the direct causal effects of the exposure on the outcome, conditional on the pleiotropic factor as a covariate included in the regression model (Burgess and Thompson, 2015). In this analysis, we included interactions between the GM/metabolites as covariates using MVMR to reduce the possible confounding variable bias (Ning et al., 2022). For MVMR analysis, the MVMR-IVW method was primarily employed.

To ensure a more rigorous interpretation of the causal relationship between GM/metabolites and PsA, multiple testing (multiple GM taxa/metabolites) was performed using the Benjamini–Hochberg FDR correction. Associations between multiple GM taxa/metabolites and PsA were considered significant when the FDR-corrected *P*-value was less than 0.05. Associations with *P*-values less than 0.05 but FDR-corrected *P*-values greater than 0.05 were considered suggestive associations between the gut microbiome/metabolites and PsA (Storey and Tibshirani, 2003).

However, Mendelian randomization (MR) estimates can introduce bias in estimating causal effects, and MR results may produce false positives when there are genetic correlations between the exposure and outcome variables (O’Connor and Price, 2018). During the process of selecting instrumental variables (IVs), it is important to note that while single nucleotide polymorphisms (SNPs) that are directly linked to the PsA outcome were eliminated, there is still a possibility that SNPs not significantly associated with PsA could still mediate the genetic risk of PsA. To address this potential issue and ensure that the causal effects were not influenced by genetic confounding or the coheritability of the exposure with the outcome, we employed the LDSC analysis. LDSC was used to evaluate the overall genetic relevance between the identified candidate gut microbiota taxa/metabolites (with an FDR less than 0.05) and PsA.

Furthermore, the bidirectional MR method was utilized to determine whether the identified GM/metabolites can be reversely affected by PsA and to identify truly potential causal GM/metabolites (Davey Smith and Hemani, 2014). Next, the causal direction of the extracted SNPs on exposures and outcomes was also assessed using MR Steiger filtering (Hemani et al., 2017). The MR Steiger filtering was used to remove SNPs that implied reverse causal direction. Through instrumenting SNPs, this method examines whether the exposure variance is greater than the outcome variance. The IVs with “TRUE” indicated causality in the expected direction, while “FALSE” implied causality reverse (Hemani et al., 2017).

The TSMR analysis was performed using the aforementioned protocol. All statistical analysis were conducted using the “TwoSampleMR,” “MVMR,” “MRPRESSO,” “LDSC software,” and “MendelianRandomization” packages (R version 4.1.2).

### 2.5 Metabolic pathway analysis and bioinformatic analysis

To further clarify the biological mechanisms underlying the candidate serum metabolites that may have causal effects on PsA at *P*_*IVW*_ < 0.05, we conducted a metabolic pathway analysis using the user-friendly web-based platform MetaboAnalyst 5.0. Specifically, we utilized its pathway analysis module^5^ (Pang et al., 2021). Metabolic Pathway analysis helps identify the pathways that contain compounds (from the input lists) which are over-represented and have significant perturbations in their concentrations. Additionally, Metabolic Pathway analysis utilizes a variety of robust statistical measures to identify which metabolites (and consequently, which pathways) are over-represented (Pang et al., 2021).

This analysis involved two major databases, namely the SMPDB and the KEGG database. The significance threshold for the metabolic pathway analysis was set at an FDR of less than 0.05.

The single nucleotide polymorphisms (SNPs) involved in the metabolic pathway were individually searched for in the QTLs database for expression quantitative trait loci (eQTLs).^6^ Novel bioinformatic analysis methods were employed to explore the functional annotation of the eQTLs involved in the metabolic pathway, including: (1) Gene Ontology (GO) enrichment analysis using the DAVID software and visualization on the Cytoscape platform; (2) Canonical pathway enrichment analysis using the KEGG^7^ and Reactome^8^ pathway databases; and (3) Construction of the Protein-Protein interaction (PPI) network using the STRING online software.^9^ A False Discovery Rate (FDR) of < 0.05 was used to filter the potentially statistically significant enriched GO terms and candidate KEGG/Reactome pathways.

### 2.6 Replication analysis

To replicate and validate our primary findings and make our results more robust, we conducted two rounds of validation analysis. (1) Replication one: We performed secondary database analysis as a replication, using another GWAS of PsA in the Immune-Mediated Inflammatory Disease Consortium as an independent validation cohort (Aterido et al., 2019). In this replication cohort study, patients with PsA were selected from the rheumatology departments of 16 Spanish hospitals. All patients with PsA were diagnosed according to the Classification Criteria for PsA. Controls were recruited from healthy blood donors from Spanish hospitals in collaboration with the Spanish DNA Bank. A case-control cohort of 835 patients with PsA and 1588 controls was finally recruited and used for GWAS. (2) Replication two: (i) Metabolites: Summary data were obtained from a GWAS of 1458 plasma metabolome in the Canadian Longitudinal Study of Aging (CLSA) as an independent validation cohort comprising 8,299 participants of European ancestry who were between the ages of 45 and 85 when recruited for biological, medical, physiological, social, lifestyle, and economic status information (Chen et al., 2023). In this study, a total of 1458 metabolites were quantified with the Metabolon HD4 platform and then batch-normalized. Only those with missing measurements in fewer than 50% of samples (*n* = 1091) were retained. (ii) GM: Data for bacterial taxa were obtained from a GWAS of 7738 individuals of European descent from the Dutch Microbiome Project (DMP). The gut microbiome was determined by shotgun metagenomic sequencing from stool samples. A total of 207 taxonomies (5 phyla, 10 classes, 13 orders, 26 families, 48 genera, and 105 species) were included in this study (Lopera-Maya et al., 2022).

## 3 Results

According to the predefined selection criteria for instrumental variables (IVs), which included SNPs that underwent clumping and harmonization, removed palindromic SNPs, and excluded SNPs with an *F*-value below 10 and the number of SNPs in each GM/metabolite less than 3, a total of 1,657 SNPs (*P* < 1 × 10^–5^) were considered as IVs associated with a total of 156 GM taxa in the MiBioGen consortium for PsA. Similarly, based on the Shin et al. (2014) Metabolic Atlas Study, we identified 7,347 SNPs as IVs associated with a total of 422 serum metabolites in the Metabolic Atlas for PsA (*P* < 1 × 10^–5^). Subsequently, the genetic variants of 12 GMs (including genus Methanobrevibacter, genus Hungatella, genus Flavonifractor, family Actinomycetaceae, genus Lachnospiraceae ND3007 group, genus Alloprevotella, genus Parabacteroides, genus Enterorhabdus, class Gammaproteobacteria, genus Eubacterium eligens group, genus Eubacterium oxidoreducens group, and genus Catenibacterium) and 37 blood metabolites [including Xanthine, X-14450, X-13477, X-12851, X-12816, X-12776, X-11850, X-11843, X-11537, X-11485, X-11452, X-06351, Valine, *Trans-*4-hydroxyproline, Stearoylcarnitine, Stachydrine, Pyroglutamylglycine, Phosphate, Phenylalanine, Linolenate (alpha or gamma; (18:3n3 or 6))], Isovalerate, HWESASXX, Homostachydrine, Homocitrulline, Histidine, Glycylvaline, Glycodeoxycholate, Fructose, Ergothioneine, Dodecanedioate, Docosahexaenoate (DHA; 22:6n3), Aspartylphenylalanine, Arabinose, ADpSGEGDFXAEGGGVR, 1-stearoylglycerophosphoinositol, 1-myristoylglycerophosphocholine, and 10-nonadecenoate (19:1n9) less than 0.5% were further excluded due to insufficient statistical power for a valid causal inference (Hemani et al., 2018). Finally, 144 GMs with 1,599 SNPs and 385 serum metabolites with 7,157 SNPs were included in this MR study. The number of IVs for each GM taxa varied from 7 to 26 SNPs, with a median of 13 SNPs, and the number of IVs for each metabolite varied from 8 to 217 SNPs, with a median of 18 SNPs, indicating a sufficient number of SNPs used in the subsequent MR analysis. The F-statistics for each GM taxa ranged from 16.91 to 88.42, with a median of 21.01, and the F-statistics for each metabolite ranged from 17.64 to 2912.88, with a median of 21.42, suggesting that there is no weak instrument bias in our MR study (Supplementary Tables 2, 3). Furthermore, the statistical power of potential eligible candidate GM/metabolites after multiple adjustments was consistently above 80%, indicating an adequate number of SNPs in this MR study.

### 3.1 Causal effects of GM on PsA

**FIGURE 2:**
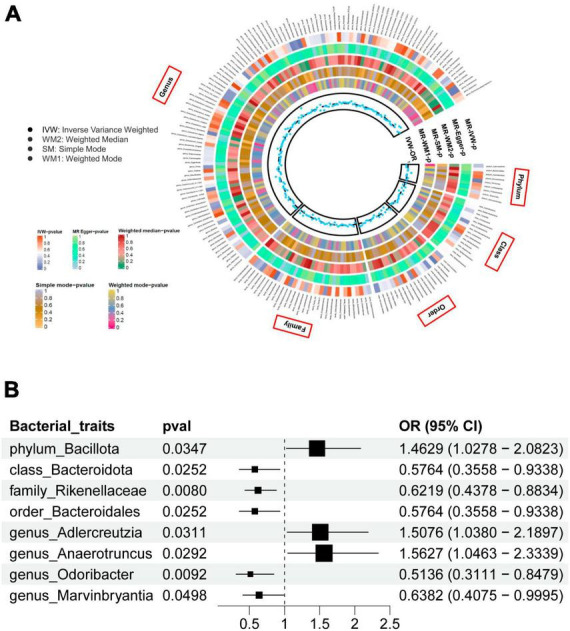
**(A)** The heatmap plot comprehensively depicts the causal analysis of gut microbiota and psoriatic arthritis. **(B)** A forest plot reveals eight bacterial traits that exhibit a significant causal link with psoriatic arthritis (IVW method, considered statistically significant with a locus-wide significance of *P* < 1 × 10^– 5^).

Gut microbiome taxa’s analysis results are shown in the heatmap plot (Figure 2A), while the significant IVW results are displayed in the forest plots (Figure 2B). In Brief, a higher genetic prediction of *Firmicutes* (*Bacillota*) at the phylum level (OR_*IVW*_: 1.463, 95% CI: 1.028–2.082, *P*_*IVW*_ = 0.031), *Adlercreutzia* at the genus level (OR_*IVW*_: 1.508, 95% CI: 1.038–2.190, *P*_*IVW*_ = 0.035), and *Anaerotruncus* at the genus level (OR_*IVW*_: 1.563, 95% CI: 1.046–2.334, *P*_*IVW*_ = 0.029) were associated with a higher risk of PsA. Conversely, a genetically predicted abundance of *Bacteroidia* (*Bacteroidota*) (OR_*IVW*_: 0.576, 95% CI: 0.356–0.934, *P*_*IVW*_ = 0.025), *Rikenellaceae* (OR_*IVW*_: 0.622, 95% CI: 0.438-0.883, *P*_*IVW*_ = 0.008), *Bacteroidales* (*Bacteroidota*) (OR_*IVW*_: 0.576, 95% CI: 0.356–0.934, *P*_*IVW*_ = 0.025), *Odoribacter* (OR_*IVW*_: 0.514, 95% CI: 0.311–0.848, *P*_*IVW*_ = 0.009), and *Marvinbryantia* (OR_*IVW*_: 0.6382, 95% CI: 0.4075–0.9995, *P*_*IVW*_ = 0.0498) were correlated with a reduced risk of PsA (Figure 2B and Supplementary Table 4). Moreover, the MR estimates of weighted median supported the significant relationships between the Genus *Anaerotruncus* and PsA, as well as the Genus *Odoribacter* and PsA (*P_weightedmedian_* < 0.05) (Supplementary Table 4).

### 3.2 Causal effects of metabolites on PsA

**FIGURE 3:**
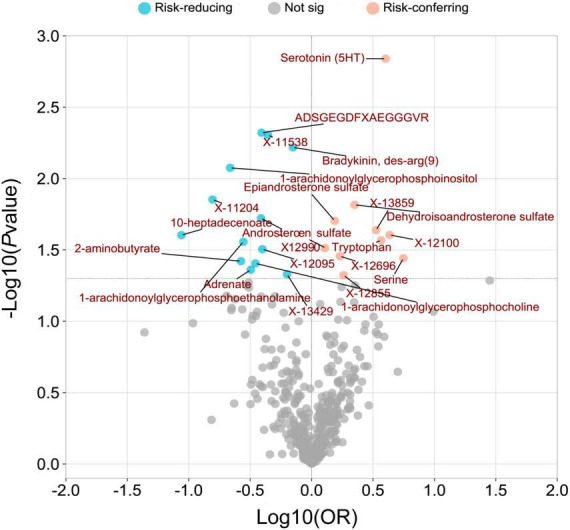
A volcano plot was created to visualize the associations between human serum metabolites and psoriatic arthritis. The plot displays the odds ratios (OR) on a log10 scale, as well as the *P*-values transformed as -log10 (*P*-value) using the inverse-variance weighted (IVW) method. The 23 metabolites that showed a causal association with psoriatic arthritis at a nominal significance level (*P*_IVW_ < 0.05) were highlighted in pink (as risk-conferring metabolites) and blue (as risk-reducing metabolites) circles.

The overall metabolic analysis results are summarized in the volcano plot (Figure 3). Among these metabolites, each SD increase in genetically determined Bradykinin, des-arg(9) (OR_*IVW:*_ 0.706, 95% CI: 0.551–0.905, *P*_*IVW*_ = 0.006), X-12095 (OR_*IVW:*_ 0.398, 95% CI: 0.172–0.921, *P*_*IVW*_ = 0.031), ADSGEGDFXAEGGGVR (OR_*IVW:*_ 0.392, 95% CI: 0.205–0.751, *P*_*IVW*_ = 0.005), X-12990 (OR_*IVW:*_ 0.388, 95% CI: 0.176–0.856, *P*_*IVW*_ = 0.019), 1-arachidonoylglycerophosphocholine (OR_*IVW:*_ 0.349, 95% CI: 0.128–0.950, *P*_*IVW*_ = 0.039), Adrenate (OR_*IVW:*_ 0.320, 95% CI: 0.106–0.968, *P*_*IVW*_ = 0.044), 1-arachidonoylglycerophosphoethanolamine (OR_*IVW:*_ 0.279, 95% CI: 0.089–0.869, *P*_*IVW*_ = 0.028), 2-aminobutyrate (OR_*IVW:*_ 0.266, 95% CI: 0.076–0.929, *P*_*IVW*_ = 0.038), 1-arachidonoylglycerophosphoinositol (OR_*IVW:*_ 0.217, 95% CI: 0.070–0.676, *P*_*IVW*_ = 0.008), and 10-heptadecenoate (OR_*IVW:*_ 0.087, 95% CI: 0.010–0.735, *P*_*IVW*_ = 0.025) was associated with a lower risk of PsA (Figure 3 and Supplementary Table 5).

While each SD increase in genetically determined Serine (OR_*IVW:*_ 5.560, 95% CI: 1.118–28.019, *P*_*IVW*_ = 0.036), X-12100-hydroxytryptophan (OR_*IVW:*_ 4.319, 95% CI: 1.203–15.502, *P*_*IVW*_ = 0.025), Serotonin (5HT) (OR_*IVW:*_ 4.009, 95% CI: 1.706–9.419, *P*_*IVW*_ = 0.001), Tryptophan (OR_*IVW:*_ 3.682, 95% CI: 1.159–11.701, *P*_*IVW*_ = 0.027), DHEA-S (OR_*IVW:*_ 2.243, 95% CI: 1.168–4.309, *P*_*IVW*_ = 0.015), Epiandrosterone sulfate (OR_*IVW:*_ 1.542, 95% CI: 1.071–2.221, *P*_*IVW*_ = 0.020), and Androsterone sulfate (OR_*IVW:*_ 1.283, 95% CI: 1.024–1.609, *P*_*IVW*_ = 0.031) was associated with a higher risk of PsA (Figure 3 and Supplementary Table 5).

The associations between 1-arachidonoylglycerophosphoe thanolamine, 1-arachidonoylglycerophosphocholine, serotonin (5HT), X-12100—hydroxytryptophan, serine, 1-arachidonoyl glycerophosphoinositol, X-11538, ADSGEGDFXAEGGGVR, X-13429, and bradykinin, des-arg (9) with PsA remained robust in at least one of three additional MR statistical methods (Supplementary Table 5).

### 3.3 Metabolic pathway and bioinformatic analysis

**FIGURE 4:**
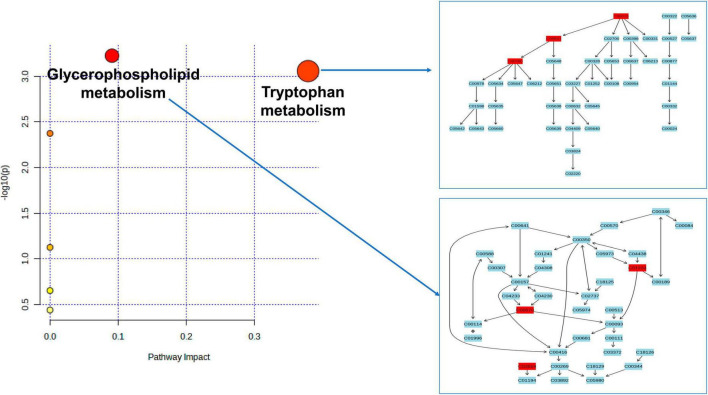
Enriched significant metabolic pathways were identified using the Benjamini-Hochberg false discovery rate (FDR) with a threshold of less than 0.05. Note: C00078: Tryptophan, C00643: hydroxytryptophan, C00780: Serotonin (5HT), C01233: 1-arachidonoylglycerophosphoethanolamine, C00670: 1-arachidonoylglycerophosphocholine, C03819: 1-arachidonoylglycerophosphoinositol.

As depicted in Figure 4 and Table 2, our analysis of metabolic pathways has revealed significant associations between the “Glycerophospholipid metabolism” pathway (*P* = 5.955E-4, FDR = 0.037) and the “Tryptophan metabolism” pathway (*P* = 8.783E-4, FDR = 0.036) with the pathogenetic process of PsA.

**TABLE 2:** Significant metabolic pathways involved in the pathogenesis of PSA.

Metabolic pathway	Match Status	Involved metabolites	*P-*value	Holm p	FDR	Databases
Glycerophospholipid metabolism	3/36	1-arachidonoylglycero phosphoinositol 1-arachidonoylglycero phosphocholine 1-arachidonoylglycero phosphoethanolamine	5.955E-4	0.050	**0.037**	KEGG
Tryptophan metabolism	3/41	Tryptophan X-12100–hydroxytryptophan Serotonin (5HT)	8.783E-4	0.073	**0.036**	KEGG SMPDB

PsA, psoriatic arthritis; KEGG, Kyoto Encyclopedia of Genes and Genomes; SMPDB, small molecule pathway database. Bold value represents the FDR <0.05.

**FIGURE 5:**
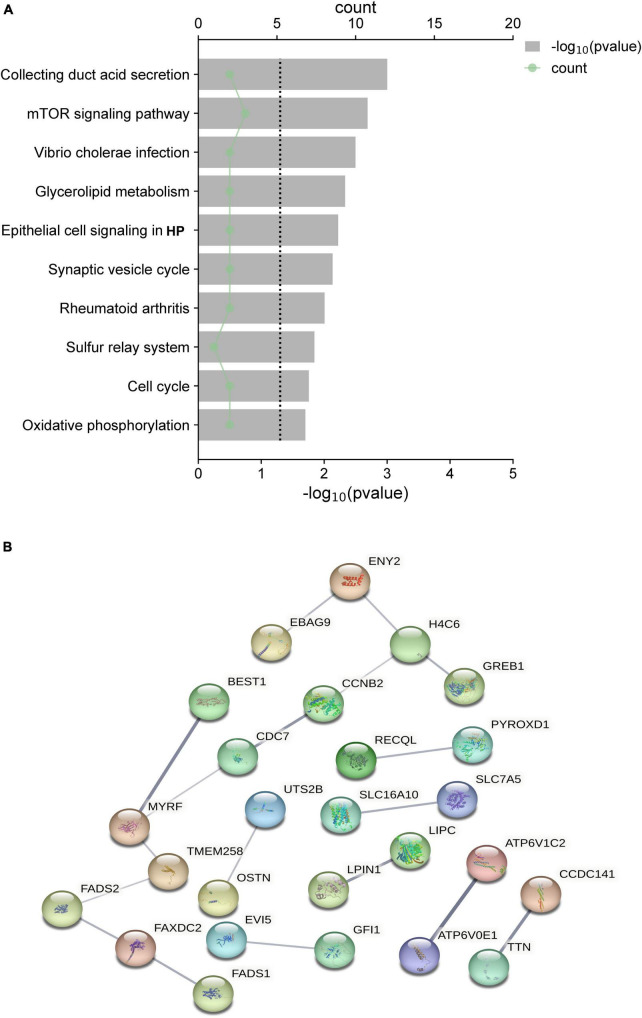
**(A)** Top 10 canonical pathways of the differential expression quantitative trait loci (eQTL) affected. **(B)** PPI network of eQTL.

**FIGURE 6:**
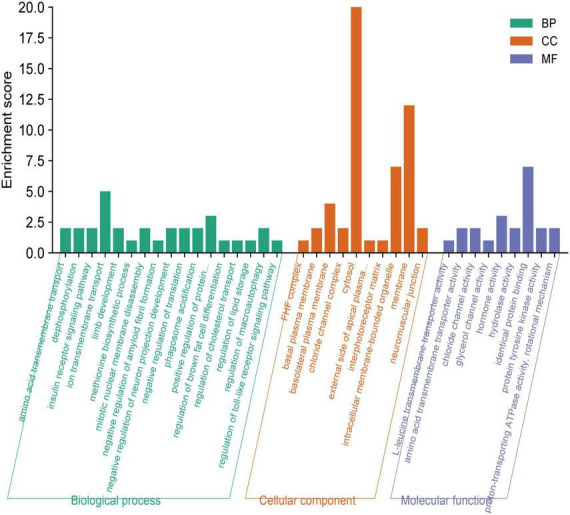
Gene ontology (GO) annotations were identified for the expression quantitative trait loci (eQTL) involved in the metabolic pathway. BP, biological function; MF, molecular function; CC, cellular component.

The functional enrichment analysis revealed that the eQTLs identified are primarily involved in Glycerolipid metabolism and the expression of key metabolic factors regulated by bacterial infections (*Vibrio cholerae* and *Helicobacter pylori*) and the mTOR signaling pathway (Figure 5A). The Protein-Protein Interaction (PPI) network of eQTLs is shown in Figure 5B. Additionally, Figure 6 summarizes the significantly enriched Gene Ontology (GO) terms for biological function, molecular function, and cellular component related to the identified eQTLs.

### 3.4 Benjamini–Hochberg FDR correction

**FIGURE 7:**
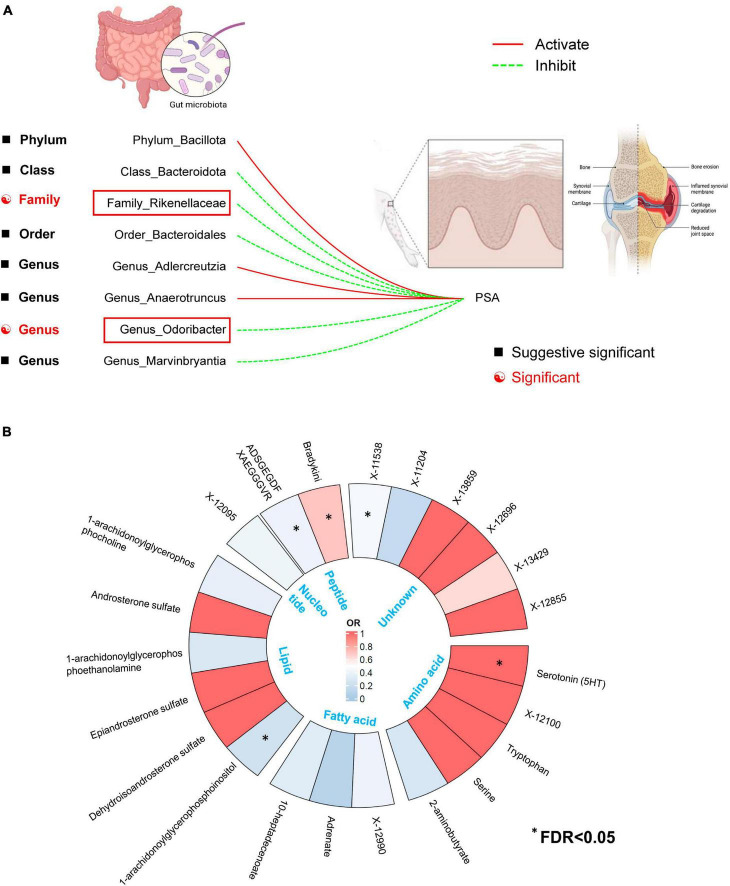
**(A)** Two out of the eight candidate intestinal bacteria were found to be significantly associated with psoriatic arthritis at a significance level corrected for the Benjamini-Hochberg false discovery rate (FDR) (IVW_FDR–corrected_ < 0.05). **(B)** Five out of the twenty-three candidate metabolites were found to be significantly associated with psoriatic arthritis at a significance level corrected for the Benjamini-Hochberg false discovery rate (FDR) (IVW_FDR–corrected_ < 0.05). *Means IVW_FDR–corrected_ < 0.05.

For the GM taxa dataset, results from the FDR correction revealed that higher relative abundances of Genus *Odoribacter* and Family *Rikenellaceae* retain significant causal relationships with a lower risk of PsA (*P*_*IVW*–FDR–corrected_ < 0.05) (Figure 7A). For the metabolic dataset, after applying this multiple-testing correction, we obtained five significant metabolite [Serotonin (5HT), ADSGEGDFXAEGGGVR, X-11538, Bradykinin, des-arg(9), 1-arachidonoylglycerophosphoinositol] associations with PsA (*P*_*IVW–FDR–corrected*_ < 0.05) (Figure 7B).

### 3.5 Genetic correlation

The results of LDSC-based estimates indicated weak overall genetic relevance between PsA and Family_*Rikenellaceae* (Rg = 0.177, *P* = 0.320), Genus_*Odoribacter* (Rg = 0.238, *P* = 0.674) Serotonin (5HT) (Rg = 0.268, *P* = 0.397), ADSGEGDFXAEGGGVR (Rg = 0.241, *P* = 0.166), X-11538 (Rg = 0.178, *P* = 0.257), and 1-arachidonoylglycerophosphoinositol (Rg = 0.246, *P* = 0.144). These results indicate that our findings are not affected by shared genetic components (Guo et al., 2023; Yun et al., 2023). Subsequently, we estimated the heritability (h^2^) of single-nucleotide polymorphisms (SNPs) in seven gut microbiota (GM) taxa/metabolites using LDSC. The h^2^ of GM taxa ranged from 0.0130 (Genus *Odoribacter*) to 0.0102 (Family *Rikenellaceae*), while the h^2^ of metabolites ranged from 0.078 (Serotonin (5HT)) to 0.1455 (1-arachidonoylglycerophosphoinositol) (Supplementary Table 6).

### 3.6 Sensitivity analysis

The outcomes from Cochran’s Q-statistic, which included the IVW test and MR-Egger regression method, showed no significant heterogeneity among these selected SNPs (*P* > 0.05) (Table 3). The MR-PRESSO test demonstrated no evidence of horizontal pleiotropy or any obvious outliers for the IVs used in this MR study (*P* > 0.05). The MR-Egger regression intercept analysis yielded similar findings (*P* > 0.05), suggesting no significant directional horizontal pleiotropy (Table 3). Finally, the leave-one-out sensitivity analysis indicated that the results of causal association between GM taxa/metabolite and PsA remained stable even after removing each SNP one by one (Supplementary Figures 1–7).

**TABLE 3:** Heterogeneity test, pleiotropy test and MR-PRESSO results of genetic variants.

Category	Heterogeneity				Pleiotropy		Pleiotropy
	**MR egger**		**IVW**		**MR egger**		**MR-PRESSO**
	**Cochran’s Q**	***P*-value**	**Cochran’s Q**	***P*-value**	**Egger intercept**	***P*-value**	**Global Test (*P*)**
**Gut microbiota**							
Genus *Odoribacter*	4.117	0.533	5.875	0.437	−0.079	0.242	0.433
Family *Rikenellaceae*	11.949	0.803	13.602	0.755	0.0481	0.216	0.788
**Metabolites**							
Serotonin (5HT)	4.517	0.991	4.991	0.992	−0.014	0.502	0.989
X-11538	3.976	0.971	4.005	0.983	0.005	0.867	0.991
ADSGEGDFXAE GGGVR	8.132	0.521	8.145	0.615	−0.003	0.912	0.715
Bradykinin, des-arg(9)	15.611	0.740	16.085	0.765	0.015	0.499	0.829
1-arachidonoylglycero phosphoinositol	21.823	0.192	22.178	0.224	0.014	0.605	0.229

### 3.7 Confounding analysis

The possible trait in each candidate GM/metabolite (*P*_*IVW–FDR–corrected*_ < 0.05) was summarized in Supplementary Table 7. There were two potentially pleiotropic SNPs (rs6029632 in ADSGEGDFXAEGGGVR metabolite and rs1260333 in 1-arachidonoylglycerophosphoinositol metabolite) identified by PhenoScanner (Table 4), and after removing these SNPs, the results were still consistent with previously significant associations [ADSGEGDFXAEGGGVR: OR_*IVW*_: 0.4030, 95% CI: 0.2076–0.7820, *P*_*IVW*_ = 0.0072; 1-arachidonoylglycerophosphoinositol: OR_*IVW*_: 0.1912, 95% CI: 0.0620–0.5899, *P*_*IVW*_ = 0.0040] (Table 5).

**TABLE 4:** Single nucleotide polymorphisms (SNPs) associated with confounding of PsA.

Category	SNP	Trait	*P-*value	Sources	References
ADSGEGDFXAEGGGVR	rs6029632	Cholesterol, total; Low density lipoprotein cholesterol levels.	2.00 E-14	Teslovich NHGRI-EBI GWAS catalog	PMID:35884923 PMID:36329257
1-arachidonoylglycero phosphoinositol	rs1260333	Total cholesterol levels; Triglyceride levels; Triglyceride levels in non-type 2 diabetes; Fasting Glucose; Renal underexcretion gout	1.22E-17	Teslovich NHGRI-EBI GWAS catalog MAGIC	PMID:26582766 PMID:31217584 PMID:20081858 PMID:32238385

**TABLE 5:** The association between genetic proxies for metabolites and PsA, after excluding pleiotropic SNPs.

Exposures	Outcome	Method	OR (95% CI)	*P-*value
ADSGEGDFXAEGGGVR	PsA	IVW	0.4030 (0.2076, 0.7820)	0.0072
1-arachidonoylglycero phosphoinositol	PsA	IVW	0.1912 (0.0620, 0.5899)	0.0040

IVW, inverse-variance weighted; PsA, psoriatic arthritis.

### 3.8 Reverse causality analysis

It is worth noting that we did not find reverse causality between these specific GM/metabolites and the risk of PsA (*P*_*IVW*_ > 0.05), which suggested the absence of reverse causality (Table 6). PsA may not be the cause of these specific GM/metabolites, but rather the outcome of them. Additionally, there was no evidence of heterogeneity and horizontal pleiotropy in the causal relationships between PsA and seven candidate GM/metabolites (*P* > 0.05) (Table 5). Next, the results of the MR Steiger test further support the causal direction from the GM/metabolite to PsA, instead of the reverse (Table 7).

**TABLE 6:** Inverse variance weighted (IVW) based MR model estimate the causal relationships between the risk of psoriatic arthritis and candidate gut microbiota/metabolites and tests for heterogeneity and horizontal pleiotropy.

Category	Effect size (IVW method)	*P-*value	Heterogeneity			Pleiotropy		MR-PRESSO
			**Method**	**Q**	***P***	**Intercept**	***P***	**Global test (*P*)**
**Gut microbiota**								
Family_Rikenellaceae	0.995 (0.971, 1.019)	0.668	IVW	6.800	0.558	0.013	0.162	0.486
			MR_Egger	4.362	0.737			
Genus_Odoribacter	1.000 (0.974, 1.028)	0.974	IVW	5.845	0.558	0.014	0.187	0.468
			MR_Egger	3.630	0.727			
**Metabolites**								
X-11538	0.994 (0.980, 1.008)	0.415	IVW	5.022	0.657	0.013	0.149	0.697
			MR_Egger	2.287	0.892			
Bradykinin, des-arg(9)	1.029 (0.994, 1.064)	0.102	IVW	4.379	0.623	−0.003	0.872	0.661
			MR_Egger	4.407	0.732			
ADSEGDFXA EGGGAR	1.007 (0.988, 1.026)	0.467	IVW	10.444	0.165	−0.007	0.578	0.205
			MR_Egger	9.876	0.130			
1-arachidonoylglycero phosphoinositol	0.996 (0.987, 1.005)	0.397	IVW	5.132	0.644	0.003	0.630	0.622
			MR_Egger	4.875	0.560			
Serotonin 5HT	0.997 (0.979, 1.016)	0.782	IVW	7.425	0.115	−0.008	0.480	0.240
			MR_Egger	4.452	0.217			

**TABLE 7:** Steiger direction test gut microbiota/metabolites to PsA.

Direction: microbiota → PsA			Direction: metabolites → PsA		
**Taxonomy**	**Direction**	**Steiger *P***	**Metabolites**	**Direction**	**Steiger *P***
Family *Rikenellaceae*	TRUE	4.50E-77	Serotonin (5HT)	TRUE	5.55E-45
Genus *Odoribacter*	TRUE	2.12E-38	X-11538	TRUE	1.34E-82
			ADSGEGDFXAEGGGVR	TRUE	1.05E-34
			1-arachidonoylglycerophosphocholine	TRUE	3.73E-82
			Bradykinin, des-arg(9)	TRUE	1.37E-106

PsA, psoriatic arthritis.

### 3.9 Multivariable MR analysis and network of identified GM features and blood metabolites interacted with PsA

**FIGURE 8:**
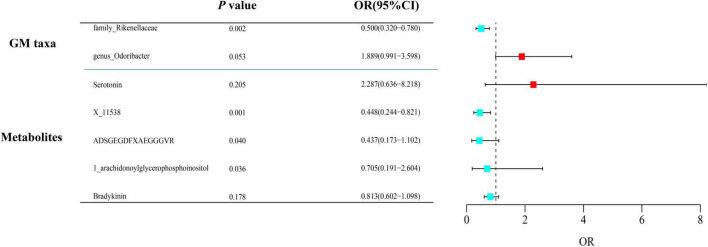
Multivariable MR analysis of the identified microbiota and metabolites. A total of 95% CI, 95% confidence interval; IVW, inverse variance weighted; MVMR, multivariable Mendelian randomization.

After adjusting for GM/metabolite interactions in MVMR analysis, one microbiota and one metabolite remained causally associated with the outcome of PsA at *P*_*MV–IVW*_ < 0.05 (Figure 8): Family *Rikenellaceae* (OR_*MV–IVW*_: 0.500, 95% CI: 0.320–0.780; *P*_*MV–IVW*_ = 0.002), and X-11538 (OR_*IVW*_: 0.448, 95% CI: 0.244–0.821, *P*_*MV–IVW*_ = 0.001). However, it should be noted that other associations may have been influenced by potential confounding variables to some extent (Figure 8). The results of MVMR_PRESSO indicated no evidence of horizontal pleiotropy in this MVMR analysis (MR-PRESSO Global Test RSSobs = 58.242, MR-PRESSO Global Test *P*-value = 0.541).

**FIGURE 9:**
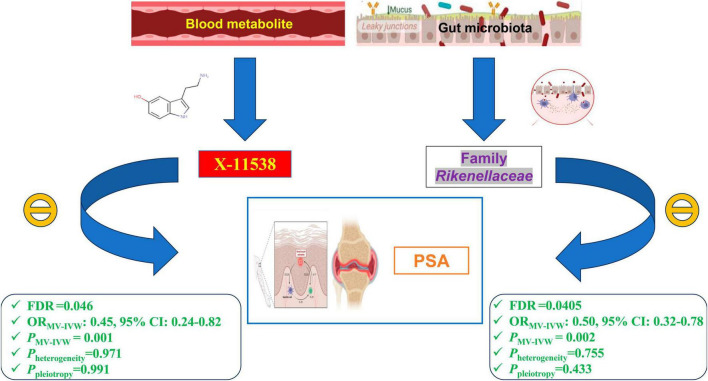
Network of identified blood metabolites and gut microbiome features interacted with psoriatic arthritis.

After obtaining potential GM taxa and metabolic candidates with a significant threshold of FDR less than 0.05 and *P*_*MV–IVW*_ < 0.05, we subsequently create a network depicting the interaction between the identified GM feature (Family *Rikenellaceae*) and the blood metabolite (X-11538) that interacts with PsA (Figure 9).

### 3.10 Replication analysis

In the first-round replication analysis (different dataset from the outcome of PsA), two GM/metabolites (Family Rikenellaceae and X-11538) were found to be significantly associated with a reduced risk of PsA. This association was observed using the instrument derived from the FinnGen Biobank cohort at an FDR of less than 0.05 and *P*_*MVMR–IVW*_ < 0.05. Additionally, the association was replicated using the instrument derived in the Immune-Mediated Inflammatory Disease Consortium of PsA at a *P*-value of less than 0.05, with the same direction of association (Table 8).

**TABLE 8:** Replication analysis to identify the association between GM/metabolite and PsA.

GM/metabolite	Data sources	Method	Effect size OR (95% CI)	*P*-value
Replication one: Replication in outcomes	Different dataset from PsA			
X-11538	Primary: PsA from FinnGen Biobank	IVW	0.44 (0.25, 0.78)	0.005
	Replication: PsA from Immune-Mediated Inflammatory Disease Consortium	IVW	0.01 (0.01, 0.72)	0.001
	**Meta-analysis**	Random Model	0.41 (0.24, 0.73)	0.002
Family *Rikenellaceae*	Primary: PsA from FinnGen Biobank	IVW	0.62 (0.44, 0.88)	0.008
	Replication: PsA from Immune-Mediated Inflammatory Disease Consortium	IVW	0.37 (0.14, 0.93)	0.035
	**Meta-analysis**	Fixed Model	0.58 (0.42, 0.81)	0.001
Replication two: Replication in exposures	Different dataset from GM/metabolites			
X-11538	Primary: X-11538 from TwinsUK and KORA cohorts	IVW	0.44 (0.25, 0.78)	0.005
	Replication: X-11538 from Canadian Longitudinal Study of Aging	IVW	0.68 (0.55, 0.86)	0.001
	**Meta-analysis**	Random Model	0.64 (0.52, 0.79)	0.001
Family *Rikenellaceae*	Primary: Family *Rikenellaceae* from MiBioGen	IVW	0.62 (0.44, 0.88)	0.008
	Replication: Family *Rikenellaceae* from Dutch Microbiome Project	IVW	0.58 (0.35,0.96)	0.033
	**Meta-analysis**	Fixed model	0.61 (0.46, 0.81)	0.001

GM, gut microbiota; IVW, inverse variance-weighted.

In the second-round replication analysis (different dataset from the exposures of GM/metabolites), two GM/metabolites (Family *Rikenellaceae* and X-11538) were detected to be significantly associated with a reduced risk of PsA at FDR < 0.05 and *P*_*MVMR–IVW*_ < 0.05 when using instruments derived either in the MiBioGen/Dutch Microbiome Project cohort (GM) or the TwinsUK and KORA/Canadian Longitudinal Study of Aging cohort (Table 8). Subsequent combined meta-analysis of different datasets further confirmed that higher levels of Family *Rikenellaceae* and X-11538 were associated with reduced risk of PsA (*P* < 0.05) (Table 8).

## 4 Discussion

To the best of our understanding, this MR study is the first to utilize comprehensive GWAS summary statistics to explore the potential causal relationship between GM/metabolite levels and the risk of PsA. Our analysis successfully identified two specific gut microbial taxa, namely Genus *Odoribacter* and Family *Rikenellaceae*, as well as five metabolites primarily involved in the peptides category of metabolites (Serotonin, ADSGEGDFXAEGGGVR, X-11538, Bradykinin, des-arg(9), and 1-arachidonoylglycerophosphoinositol). Importantly, these correlations were found to be causal in nature, even after adjusting for multiple testing using the FDR method (all with IVW_*FDR–corrected*_ < 0.05) (Figure 7). Additionally, our findings revealed that the Glycerophospholipid metabolism pathway and the Tryptophan metabolism pathway are crucial in the pathogenesis of PsA (FDR < 0.05) (Figure 4). The functional enrichment analysis revealed that the eQTLs examined were primarily associated with glycerolipid metabolism and the expression of key metabolic factors influenced by bacterial infections (*Vibrio cholerae* and *Helicobacter pylori*) as well as the mTOR signaling pathway. It is interesting to note, however, that the effects of these associations were weakened in our MVMR analyses, which incorporated adjustment for gut microbiota/metabolite interactions (Figure 6). As a result, only one microbiota (Family *Rikenellaceae*) and one metabolite (X-11538) remained as significant protective factors for the development of PsA (*P*_*MV–IVW*_ < 0.05) (Figure 8). Overall, these findings provide valuable insights into the complex relationship between GM/metabolite and PsA, specifically within the gut microbiota-joint axis.

*Firmicutes* (*Bacillota*) and *Bacteroidetes* (*Bacteroidota*) make up the majority of the gut microbiota in humans, accounting for approximately 90% (Rinninella et al., 2019). Numerous observational studies have shown disruptions in the microbial composition of *Firmicutes* (*Bacillota*) and *Bacteroidetes* (*Bacteroidota*). For example, a study compared the microbial composition of fecal samples from individuals with psoriatic disorder to that of healthy controls who were matched in terms of age, BMI, and gender (Chen et al., 2018). The findings revealed an increase in *Firmicutes* (*Bacillota*) and a decrease in *Bacteroidetes* (*Bacteroidota*) concentrations in psoriatic subjects. Furthermore, the Firmicutes (*Bacillota*)/Bacteroidetes (*Bacteroidota*) (F/B) ratio in psoriatic patients was more than twice that of the healthy controls who were matched for age and BMI (Shapiro et al., 2019). Another study conducted by Doaa et al. (2016) found a positive correlation between the F/B ratio in psoriasis patients and disease severity as measured by the PASI score. In a study by Yeh et al. (2019), it was discovered that the FDA-approved drug secukinumab, a human anti-Th17 monoclonal antibody, could reduce inflammation and improve physical activity in PsA patients by upregulating the expression of *Bacteroidia* (*Bacteroidota*) and downregulating the abundance of *Firmicutes* (*Bacillota*). Our research findings support the results of previous observational studies and provide additional evidence of a positive association between the relative abundance of *Firmicutes* (*Bacillota*) and the risk of PsA. Conversely, we found a negative relationship between the relative abundance of Class *Bacteroidia* (*Bacteroidota*), its subordinate Order *Bacteroidales*, and the risk of PsA, as shown in Figure 2. Furthermore, it was observed that the *Firmicutes* (*Bacillota*)*/Bacteroidota* (*Bacteroidota*) ratio served as an important biomarker for assessing gut disturbance and gut microbiota status not only in PsA patients but also in those with comorbidities like non-alcoholic fatty liver disease (Sobhonslidsuk et al., 2018), obesity (Stojanov et al., 2020), insulin resistance (Moreno-Indias et al., 2016), and cardiovascular disease (CVD) (Magne et al., 2020). The disturbance in the *Firmicutes (Bacillota)-to-Bacteroidetes* ratio, also known as the F/B ratio, indicates an imbalance in the gut bacterial epithelium’s integrity. As a result, there is an increase in gut permeability, which causes the gut to leak, potentially allowing bacteria to move from the gut lumen into the systemic circulation. These processes can activate and stimulate the immune response, disrupt T cell regulation, and encourage bacterial chemotaxis. This series of events can trigger the production of pro-inflammatory cytokines, leading to chronic low-grade systemic inflammation. This inflammation directly impacts the condition of the skin and joints, eventually leading to the development of psoriatic skin issues and physical disability in individuals with PsA. However, a clinical study conducted by Tan et al. (2018) detected an increased relative abundance of *Bacteroidaceae* in psoriatic patients compared to healthy controls. The different taxa of *Bacteroidota* in the gut microbiota have conflicting roles. Some *Bacteroidota*, known as enterotoxic *Bacteroides*, impair the gut barrier and release inflammatory cytokines into the bloodstream, while others, referred to as non-toxigenic *Bacteroides* (*Bacteroidota*), have an anti-inflammatory effect by producing short-chain fatty acids (Valguarnera and Wardenburg, 2020). Therefore, the conflicting results from previous observational studies, as well as this MR study, highlight the need for a more comprehensive classification of gut microbiota at the taxonomy level.

A decrease in the abundance of Genus_*Odoribacter* has been observed in patients suffering from rheumatoid arthritis (Sun et al., 2019) and colitis (Zhang et al., 2020). Conversely, an increase in the abundance of this strain has been identified in individuals with Spondyloarthritis (González-Chávez et al., 2023). Additionally, the combined treatment with glucosamine and chondroitin sulfate has shown potential in improving rheumatoid arthritis in rats by effectively regulating the relative abundance of Genus_*Odoribacter* (Wang et al., 2023b). This study found a strong correlation between a high abundance of Genus_*Odoribacter* and a decreased risk of PsA, a relationship that was further confirmed through multiple testing and MVMR analysis. However, it is important to note that no previous observational study has directly reported an association between the relative abundance of Genus_*Odoribacter* and the risk of PsA. Consequently, cautious interpretation of these findings is warranted.

Over the past few years, there have been several studies conducted utilizing both human and rodent models in order to investigate the connection between the relative abundance of *Rikenellaceae* and PsA. PsA is classified under a specific group known as spondyloarthropathies (SpA). In a transgenic rat model with HLA-B27, it was observed that a higher presence of *Rikenellaceae* in both the lumen and mucosa of the gut was associated with a reduction in gut inflammation and improved symptoms of arthritis. This finding was consistent with a clinical study conducted by Hidalgo-Cantabrana et al. (2019). In their research (Hidalgo-Cantabrana et al., 2019), it was noted that the relative abundance of *Rikenellaceae* at the family level was decreased in psoriatic patients when compared to healthy individuals. However, conflicting results were discovered in a study conducted by Costello et al. (2015), which found an increase in *Rikenellaceae* in PsA patients based on microbiome analysis of terminal ileum biopsy specimens. It is likely that these inconsistent results can be attributed to differences in sample collection methods for microbiome analysis. Hidalgo-Cantabrana et al. (2019) utilized fecal samples, while Costello et al. (2015) used terminal ileum biopsy specimens for 16S rRNA gene sequencing. Furthermore, the previous association between gut microbiota and PsA may have been influenced by confounding factors. However, research utilizing Mendelian Randomization (MR) methods can help reduce the impact of these confounding variables in comparison to previous observational clinical studies. According to the current findings from this MR study, which were confirmed via MVMR analysis, it was revealed that the relative abundance of the Family *Rikenellaceae* was decreased in PsA patients in comparison to healthy controls, supporting the results observed by Hidalgo-Cantabrana et al. (2019). However, further clarification is required regarding the inverse relationship between the Family *Rikenellaceae* and the risk of PsA. This necessitates a comprehensive longitudinal study and exploration of the potential biological role of the Family *Rikenellaceae* in the mechanisms of PsA in future research endeavors.

According to the findings from the metabolic pathway analysis presented in Figure 4 and Table 2, it was observed that the Tryptophan metabolism pathway (which includes tryptophan, hydroxytryptophan, and serotonin) (FDR = 0.036) and the Glycerophospholipid metabolism pathway (which includes 1-arachidonoylglycerophosphoethanolamine, 1-arachidonoylglycerophosphocholine, and 1-arachidonoylglycerophosphoinositol) (FDR = 0.037) played significant roles in the potential metabolic mechanisms of PsA. One of the key metabolites mentioned in relation to the hyperactivity of the tryptophan metabolism pathway was tryptophan, which was found to increase the risk of PsA in our MR research. This finding is consistent with a prior large-scale observational study, which reported high levels of tryptophan in the serum and synovial fluids of PsA patients compared to patients with rheumatoid arthritis (Bertazzo et al., 1999). In addition, the results of our MR research are also in line with previous longitudinal and *in vivo* studies. For instance, Chen et al. (2021) conducted a study using non-targeted LC-MS-based metabolomics and discovered that several essential amino acids, including tryptophan, were significantly upregulated in plasma samples from psoriatic patients. They further observed that tryptophan was strongly associated with disease activity in psoriatic disorders, even after adjusting for confounders such as age, sex, and BMI. Similar findings have been supported by other studies, and *in vivo* experimental studies have revealed that disturbances in the tryptophan metabolic pathway, along with changes in tryptophan and hydroxytryptophan levels, contribute to oxidative stress, T cell activation, and the release of pro-inflammatory cytokines in a rat model of spondyloarthritis (Asquith et al., 2017; Xu et al., 2021, 2022). Interestingly, Aylward and Maddock (1974) reported higher serum concentrations of tryptophan in patients with PsA when they discontinued their anti-rheumatic medications for a week. However, these levels significantly decreased when patients resumed their regular medication. These findings are consistent with a case study where a PsA patient experienced considerable improvement in arthritis symptoms by restricting their dietary intake of tryptophan (Auckland, 1969). Thus, the results of our MR study, along with previous observational studies, indicate a positive association between elevated levels of tryptophan and hydroxytryptophan and an increased risk of PsA. It is crucial to give special attention to the patient’s diet, avoiding foods that are high in tryptophan, and ensuring regular use of anti-rheumatic drugs.

Serotonin is synthesized from tryptophan and acts as a neurotransmitter, affecting neurons and potentially influencing pain, mood, and the sleep-wake cycle in patient (Müller and Schwarz, 2007). Existing literature suggests that individuals with spondyloarthritis and chronic widespread pain (Müller and Schwarz, 2007), as well as those with established rheumatoid arthritis (Kopp and Alstergren, 2002; Klavdianou et al., 2016), often have high levels of serotonin. Furthermore, acupuncture treatment at the *Zusanli* acupuncture point for a 20-min session has been shown to increase pain thresholds in arthritis model rats by releasing serotonin associated with mast cells located in acupuncture points (Li et al., 2023). However, there is currently limited evidence regarding the relationship between serotonin and the risk of PsA. In this study, we observed that elevated serotonin levels were indeed a risk factor for PsA, and these findings were further confirmed through multiple test correction (IVW_FDR–corrected_ *P* = 0.033) (Figure 4B). A previous animal study has suggested that serotonin may influence psoriatic arthritis by regulating the balance between Th17 cells and T-regulatory cells, as well as osteoclastogenesis. However, further investigation is needed to fully understand the exact mechanism involved.

Previously, Ambrożewicz et al. (2018) reported a significant decrease in glycerophospholipid molecules in PsA patients when compared to healthy controls, while Looby et al. (2021) reported opposite results for PsA patients. The discrepancies in these clinical observational studies may be attributed to differences in sample heterogeneity (age ranges of 17–66 vs. 47–69 years), sample sizes (68 vs. 40), and the use of different high-throughput metabolomics sequencing techniques (Hydrophilic interaction liquid chromatography (HILIC-LC)-MS vs. SPME coupled to LC-HRMS). In our MR study, we focused on three specific glycerophospholipid metabolism-associated metabolites, particularly 1-arachidonoylglycerophosphoinositol, which we confirmed through multiple-testing correction methods. These metabolites were found to be downregulated in PsA patients compared to the healthy controls, consistent with the findings of Ambrożewicz et al. (2018) and supported by another metabolomic study by Ottas et al. (2017). Considering the limitations of previous observational studies, such as small sample sizes and potential confounding factors, the association between glycerophospholipid and the identified metabolites in our MR analysis may be more specific and robust. In addition, our previous research conducted on mice with arthritis supports the notion that an alteration in glycerophospholipid metabolism can potentially activate neutrophils, leading to the production of pro-inflammatory cytokines. Consequently, this cascade ultimately induces oxidative stress and subsequent damage to the cell membrane (Xu et al., 2021; Xu et al., 2022).

In bioinformatics analysis, bacterial infections (*Vibrio cholerae* and *Helicobacter pylori*) and mTOR signaling pathways may mediate the glycerol lipid metabolism process involved in PsA. Several studies have suggested a potential link between bacterial infection and the development or exacerbation of arthritis. For example, a study by Tuompo et al. (2020) found that patients with reactive arthritis had a higher prevalence of gastrointestinal infections, including those caused by *Vibrio cholerae*, compared to healthy controls. Furthermore, the study reported that individuals with reactive arthritis who had a history of *Vibrio cholerae* infection exhibited more severe joint symptoms and higher levels of inflammation (Yong et al., 2018). Additionally, a recent meta-analysis found a significant association between *H. pylori* infection and the risk of developing PsA. Furthermore, research on the role of gut microbiota in the pathogenesis of PsA has raised the possibility that bacterial infection may disrupt the microbiome and contribute to the development of PsA (Scher et al., 2015). Given the potential implications for both treatment and prevention, further investigation into the relationship between *Vibrio cholerae*, *Helicobacter pylori*, and PsA is warranted. The mTOR pathway is a central regulator of cell growth, proliferation, and metabolism, and has been shown to play a crucial role in the pathogenesis of various autoimmune and inflammatory diseases, including PsA (Suto and Karonitsch, 2020). Dysregulation of mTOR signaling has been implicated in the abnormal proliferation of synoviocytes, enhanced production of proinflammatory cytokines, and aberrant differentiation of T cells in rheumatic conditions (Benham et al., 2015). Furthermore, mTOR inhibitors have demonstrated efficacy in the treatment of psoriasis and other autoimmune diseases, highlighting the importance of this pathway in the pathogenesis of PsA (Kaplan et al., 2014; Raychaudhuri and Raychaudhuri, 2014).

ADSGEGDFXAEGGGVR is a unique peptide responsible for cleaving fibrinogen, a well-established pleiotropic protein implicated in various inflammatory responses (Weisel, 2005). It has been observed that individuals with PsA have a higher risk of cardiovascular mortality and morbidity (Kimhi et al., 2007). Several studies (Ernst and Resch, 1993; Peters et al., 2004) have indicated that fibrinogen can serve as a valuable biomarker in predicting major cardiovascular risks in PsA patients, even after considering factors such as age, gender, and hypertension. In this particular MR study, we discovered a negative correlation between the levels of ADSGEGDFXAEGGGVR metabolite and the risk of PsA. This finding aligns to some extent with a comprehensive prospective epidemiologic study conducted by Stolzenberg-Solomon et al. (2020), which indicated a protective association between ADSGEGDFXAEGGGVR and the risk of pancreatic cancer. However, it is important to note that no clinical or *vivo* studies have provided direct evidence supporting the protective association between ADSGEGDFXAEGGGVR and the risk of PsA. Therefore, it is crucial to approach these results with caution. Additionally, further investigation is required to explore the potential mechanism behind the cardiac protective effect of ADSGEGDFXAEGGGVR in PsA.

Bradykinin-des-arg(9) is a metabolite derived from bradykinin, and has emerged as a promising biomarker for metabolic syndrome (MetS) (Cyr et al., 2001; Sugawara et al., 2021). The presence of a strong association between MetS and PsA has been firmly established in several studies (Husted et al., 2011; Jafri et al., 2017), with 24–58% of PsA patients also exhibiting MetS (Mok et al., 2011; Labitigan et al., 2014). Moreover, it has been observed that the components of MetS often manifest prior to the onset of PsA, with a time lag of at least 5 years (Kristensen et al., 2017). Notably, the heightened cardiovascular risk observed in PsA could be attributable to the presence of MetS. Hence, the use of Bradykinin-des-arg(9) as a novel metabolic biomarker may hold promise for monitoring cardiovascular risk in patients with PsA. However, just like ADSGEGDFXAEGGGVR, the current body of literature does not present conclusive proof surrounding the established link between Bradykinin-des-arg(9) and the likelihood of PsA. Therefore, it is crucial to exercise caution when interpreting the inverse association between metabolic levels and the risk of PsA as indicated by our MR research, while also considering the existing information. X-11538, an unidentified metabolite, exhibited a significant inverse correlation with the risk of PsA, even when accounting for multiple testing correction and using multivariable MR analysis. Notably, our MR findings offer valuable insights into the potential association between unknown metabolites and the susceptibility to PsA.

However, several limitations should be acknowledged. Firstly, it should be noted that the majority of participants in the GWAS were of European descent. Secondly, the use of 16S rRNA gene sequencing in the MiBioGen consortium’s GWAS data on gut microbiota only allows for the detection of genetic data at the genus to phylum level. This means that more specialized information at the species or strain level may be lacking in this study. Thirdly, due to the insufficient number of SNPs available for subsequent MR analysis in the MiBioGen consortium, we opted to include only GM-related SNPs at a slightly higher threshold (1 × 10^–5^) instead of the conventional threshold (5 × 10^–8^). This decision was based on a prior publication (Su et al., 2023). However, we did exclude SNPs with F-statistics below 10 to minimize the potential for weak instrument bias and improve the overall robustness of the statistical results (Burgess and Thompson, 2011). Fourthly, after correcting for multiple testing and MVMR analysis, we only observed a significant causal relationship between a specific GM feature (Family *Rikenellaceae*) and metabolite (X-11538) with PsA. Fifthly, it is worth noting that the composition of gut microbiota and metabolites can be influenced by various environmental factors, such as gender, ethnicity, disease activity, and the usage of immunosuppressive agents. Unfortunately, the original studies did not provide detailed demographic data, making it unfeasible to perform additional subgroup analysis until now. Lastly, multi-omics datasets, combined with clinical information for independent validation, could be considered as a reasonable method to replicate this current MR results. We made every effort to search relevant public databases containing both GM and metabolite datasets along with clinical information. We utilized four validated GM/metabolites databases: Metabolomicsworkbench,^10^ GMrepo,^11^ GutMega,^12^ and GutMDisorder^13^ to identify potential GM/metabolites for PsA. Unfortunately, we only were able to access multi-omics datasets (GM/metabolites) with clinical information for the outcome of psoriasis, rather than for PsA. The UK Biobank may have more GM/metabolite datasets with clinical information for PsA. However, our research team has not been able to obtain permission from the UKB due to lack of funding support and the limited study period. As a result, consistent with several previously published GM/metabolite-related MR studies (Wang et al., 2023a; Zhang X. et al., 2023; Zhang Z. Y. et al., 2023; Zhong et al., 2024), we were unable to replicate the findings of GM/metabolite-related MR studies using multi-omics data from clinical research datasets. Our study follows the same replication strategy as previous GM/metabolite-related MR studies, incorporating exposure (GM/metabolites) or outcome (PsA) from different cohorts and conducting a meta-analysis on the results. Currently, our research group is in the process of applying for relevant funds and ethical approvals to conduct multi-omics clinical research on PsA. We believe that our future results from multi-omics datasets with clinical information for PsA will further validate the results of this MR study.

Despite the limitations mentioned above, our study possesses several notable advantages. Firstly, as far as we are aware, this MR study is the first of its kind to explore the potential link between GM/metabolites and the risk of PsA. This signifies a significant contribution to the existing body of knowledge in this field. Additionally, our study capitalizes on the utilization of large publicly available GWAS datasets, which reinforces the validity and generalizability of our findings. Specifically, we accessed data from the MiBioGen consortium (GM: *N* = 18,340), TwinsUK and KORA cohorts (Metabolites: *N* = 7,824), and FinnGen (PsA: *N* = 339,050). By employing this MR study design, we effectively mitigate the issues of inconsistent selection bias and reverse causation that commonly plague traditional epidemiological studies (Smith and Ebrahim, 2003). Furthermore, in order to enhance the robustness and replicability of our findings, we conducted a sensitivity analysis known as MVMR. This analysis allowed us to account for potential interactions between the GM and metabolite variables. Ultimately, this approach enabled us to establish the significance of the GM feature (Family *Rikenellaceae*) and the metabolite (X-11538) as they relate to PsA. Lastly, although previous observational studies have conducted preliminary explorations into the relationship between GM/metabolites and PsA, there are still limitations, such as the limited number of GM/metabolites detected and the presence of contradictory results. The findings of this Mendelian randomization study further enhance the understanding of previously unidentified metabolites and GM, and validate the controversial GM and metabolic molecules from previous research.

## 5 Implication for clinical practice

Our findings have some important clinical implications. Diagnosing PsA can be challenging due to its often non-specific symptoms and the lack of a diagnostic biomarker. The Classification Criteria for Psoriatic Arthritis (CASPAR) criteria, established in 2006, have been widely used for classifying PsA in clinical trials and research studies (Chandran et al., 2007). However, recent studies have identified limitations in the CASPAR criteria, particularly in accurately identifying PsA patients with early or mild disease (Coates et al., 2016). Moreover, challenges persist when using the CASPAR criteria in dermatology or rheumatology clinics. For example, changes in finger (toe) nails are often misdiagnosed as onychomycosis, while early symptoms of joint swelling and pain are frequently misdiagnosed as rheumatoid arthritis or osteoarthritis (Hetland, 2020). Most psoriatic skin lesions of PsA manifest earlier than joint lesions with various types, such as vulgar, pustular, or erythematous. Acute phase reactants such as ESR and CRP are often normal despite active disease (FitzGerald et al., 2021). Research has shown that the delayed identification and treatment of PsA can lead to lasting damage to the joints and decreased mobility (Ciccia et al., 2017). Therefore, there is a significant need to identify new diagnostic biomarkers in order to more accurately monitor the early development of PsA.

It is important to note that we did not find evidence of reverse causality between the Family *Rikenellaceae*/X-11538 and PsA. This suggests that PsA may not be the cause of these intestinal flora/metabolic disorders, but rather a result of them. Our study established direct and reverse causal links between the Family *Rikenellaceae*/X-11538 and PsA, something that previous studies had not done. This could help in identifying ways to use specific microbiota (Family *Rikenellaceae*) and metabolite (X-11538) as useful biomarkers for early disease detection in PsA. It is also worth mentioning that with advancements in metagenomic sequencing, 16S rRNA sequencing, ultra-performance liquid chromatography (UPLC) coupled with mass spectrometry technology, detecting the abundance of Family *Rikenellaceae* and serum levels of X-11538 is not difficult. Therefore, monitoring the levels of Family *Rikenellaceae* and X-11538 would benefit the early prediction of PsA.

Current treatment options for PsA are limited, with non-steroidal anti-inflammatory drugs (NSAIDs), disease-modifying antirheumatic drugs (DMARDs), and biologic agents being the mainstays of therapy (Ogdie et al., 2020). NSAIDs are commonly used to manage pain and inflammation in PsA, but they do not alter the long-term progression of the disease. DMARDs, such as methotrexate and sulfasalazine, have been utilized for their disease-modifying effects, but their efficacy is variable, and they may have significant side effects (Ciccia et al., 2017). Biologic agents, such as TNF inhibitors and IL-17 inhibitors, have revolutionized the treatment of PsA by targeting specific inflammatory pathways; however, not all patients respond to these agents, and many experience a loss of response over time (FitzGerald et al., 2021).

Some comprehensive interventions, such as fecal microbiota transplantation (FMT), have demonstrated promising joint protective effects and reduced the accumulation of uremic toxins in recent clinical trials. A recent exploratory randomized placebo-controlled trial by Kragsnaes et al. (2021) investigated the safety and efficacy of FMT for active peripheral PsA in Denmark (NCT03058900). The study found that FMT was well-tolerated and resulted in significant improvements in clinical outcomes, including joint pain and swelling, compared to the placebo group. These findings suggest that FMT may be a promising treatment approach for active peripheral PsA, highlighting the need for further investigation into its potential mechanisms and long-term effects. Moreover, two clinical case reports from China (Yin et al., 2019) and Australia (Selvanderan et al., 2019) also proved that FMT could reduce the disease activity for patients with PsA. In this Mendelian Randomization (MR) study, the Family *Rikenellaceae* was identified as a significant protective factor for the development of PsA (OR_IVW_: OR_IVW_: 0.622, 95% CI: 0.438–0.883). This finding suggests that targeting the gut microbiota, specifically by using a selected microbiota from the *Rikenellaceae* family, may hold promise for the treatment of PsA. Therefore, further investigation into the potential therapeutic effects of FMT using a selected gut microbiota from the *Rikenellaceae* family for the treatment of PsA is warranted. Furthermore, the X-11538 metabolite also demonstrated a negative causal relationship with PsA in this MR study (OR_IVW_: 0.442, 95% CI: 0.250–0.781). Given the limited therapeutic options for PsA, it is clinically relevant for rheumatologists and dermatologists to consider X-11538 as a potentially effective supplement in the prevention and alleviation of PsA. However, further clinical trials are required to confirm the effective dose of X-11538 in managing PsA. Hence, gaining insight into the fundamental interactions between GM/metabolite and PsA has the potential to drive the creation of precise and innovative drug treatments for PsA. This could not only aid in avoiding the adverse effects linked to NSAIDs and DMARDs but also significantly accelerate the progress of personalized medicine for PsA.

Overall, as precision medicine targeting the “gut-joint” axis continues to evolve, a more comprehensive understanding of complex relationships between the gut microbiota and metabolites and PsA will pave the way for novel diagnostic and therapeutic strategies for PsA and other autoimmune rheumatic diseases.

## 6 Conclusion

In conclusion, our study demonstrates that Family *Rikenellacea*e and X-11538 exhibit a strong and negative causal relationship with PsA. These particular GM taxa and metabolites have the potential to serve as innovative biomarkers, offering valuable insights into the treatment and prevention of PsA. Moreover, bacterial infections and mTOR-mediated activation of metabolic factors may play an important role in this process.

## Data availability statement

The datasets presented in this study can be found in online repositories. The names of the repository/repositories and accession number(s) can be found in the article/Supplementary material.

## Author contributions

XX: Data curation, Formal analysis, Methodology, Resources, Writing – original draft. L-yW: Data curation, Formal analysis, Methodology, Resources, Writing – original draft. S-yW: Validation, Visualization, Writing – original draft. MY: Validation, Visualization, Writing – review and editing. Y-HW: Validation, Visualization, Writing – original draft, Writing – review and editing. LL: Validation, Visualization, Writing – review and editing. Z-lS: Software, Validation, Writing – review and editing. J-XZ: Project administration, Supervision, Writing – review and editing.

## Footnotes

1
www.mibiogen.org

2
https://gwas.mrcieu.ac.uk/

3
https://sb452.shinyapps.io/power

4
http://www.phenoscanner.medschl.cam.ac.uk/

5
https://new.metaboanalyst.ca/MetaboAnalyst/upload/PathUploadView.xhtml

6
http://www.mulinlab.org/qtlbase/index.html

7
https://www.kegg.jp/

8
https://reactome.org/

9
https://www.string-db.org/

10
https://www.metabolomicsworkbench.org/

11
https://gmrepo.humangut.info/home

12
http://gutmega.omicsbio.info/

13
http://bio-annotation.cn/gutMDisorder/
