# Supplementary material for: Investigating causal associations among gut microbiota, metabolites, and psoriatic arthritis: a Mendelian randomization study
Source: Front Microbiol. 2024 Feb 15;15:1287637. doi: 10.3389/fmicb.2024.1287637 (PMC10902440; doi:10.3389/fmicb.2024.1287637)
Supplement: Supplementary file 2 [file Data_Sheet_1.ZIP › supplementary_figures/Figure_caption.docx]

Supplementary Figure S1. Leave-one-out sensitivity analysis for Family Rikenellaceae.

Supplementary Figure S2. Leave-one-out sensitivity analysis for Genus Odoribacter.

Supplementary Figure S3. Leave-one-out sensitivity analysis for Serotonin.

Supplementary Figure S4. Leave-one-out sensitivity analysis for X-11538.

Supplementary Figure S5. Leave-one-out sensitivity analysis for ADSGEGDFXAEGGGVR.

Supplementary Figure S6. Leave-one-out sensitivity analysis for Bradykinin.

Supplementary Figure S7. Leave-one-out sensitivity analysis for 1-arachidonoylglycerophosphoinositol.
