# Supplementary figures and images for: Investigating causal associations among gut microbiota, metabolites, and psoriatic arthritis: a Mendelian randomization study
Source: Front Microbiol. 2024 Feb 15;15:1287637. doi: 10.3389/fmicb.2024.1287637 (PMC10902440; doi:10.3389/fmicb.2024.1287637)

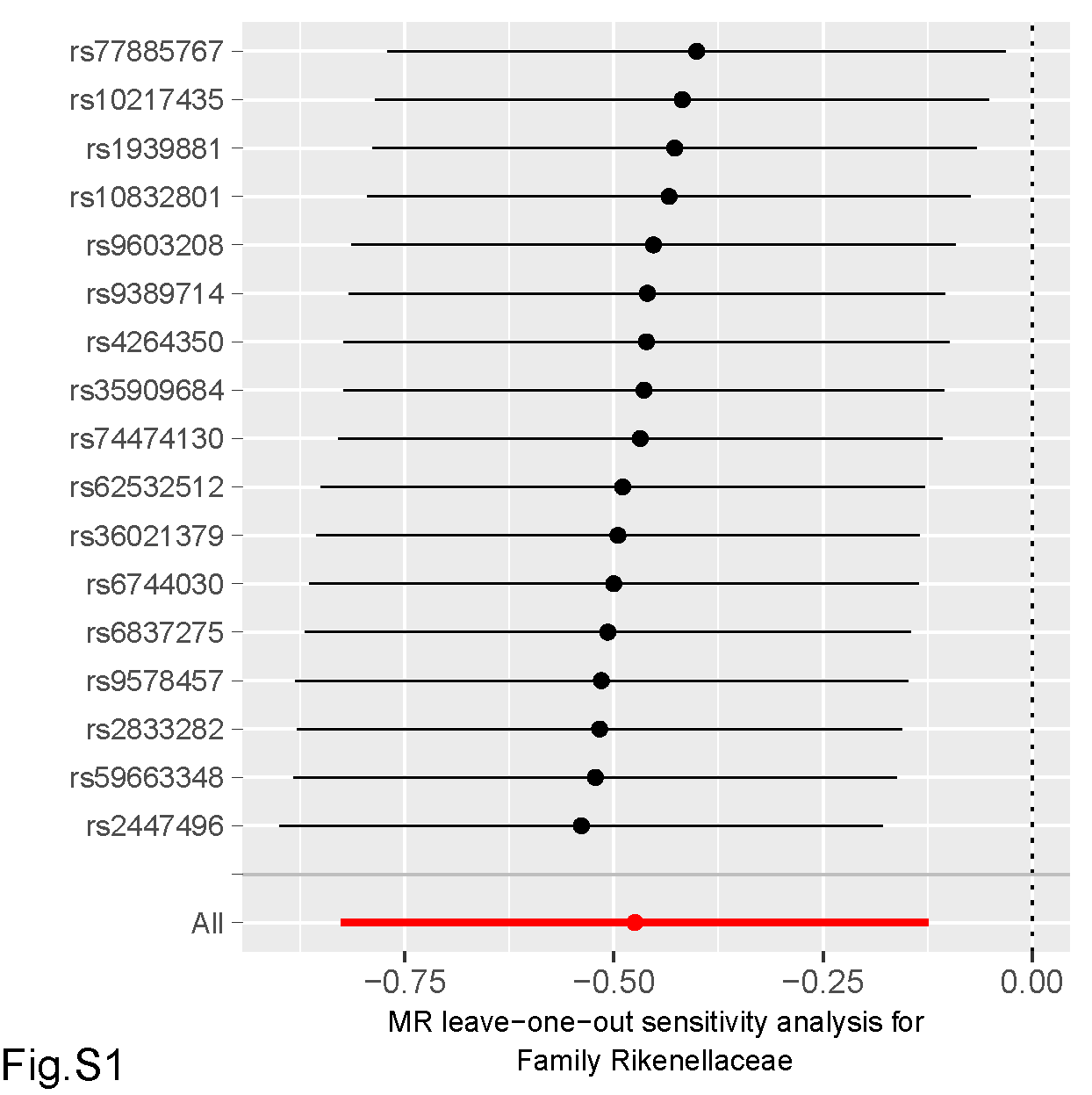

Supplement: Supplementary file 2 [file Data_Sheet_1.ZIP › supplementary_figures/Fig.S1_Family_Rikenellaceae_Rplot.tif]

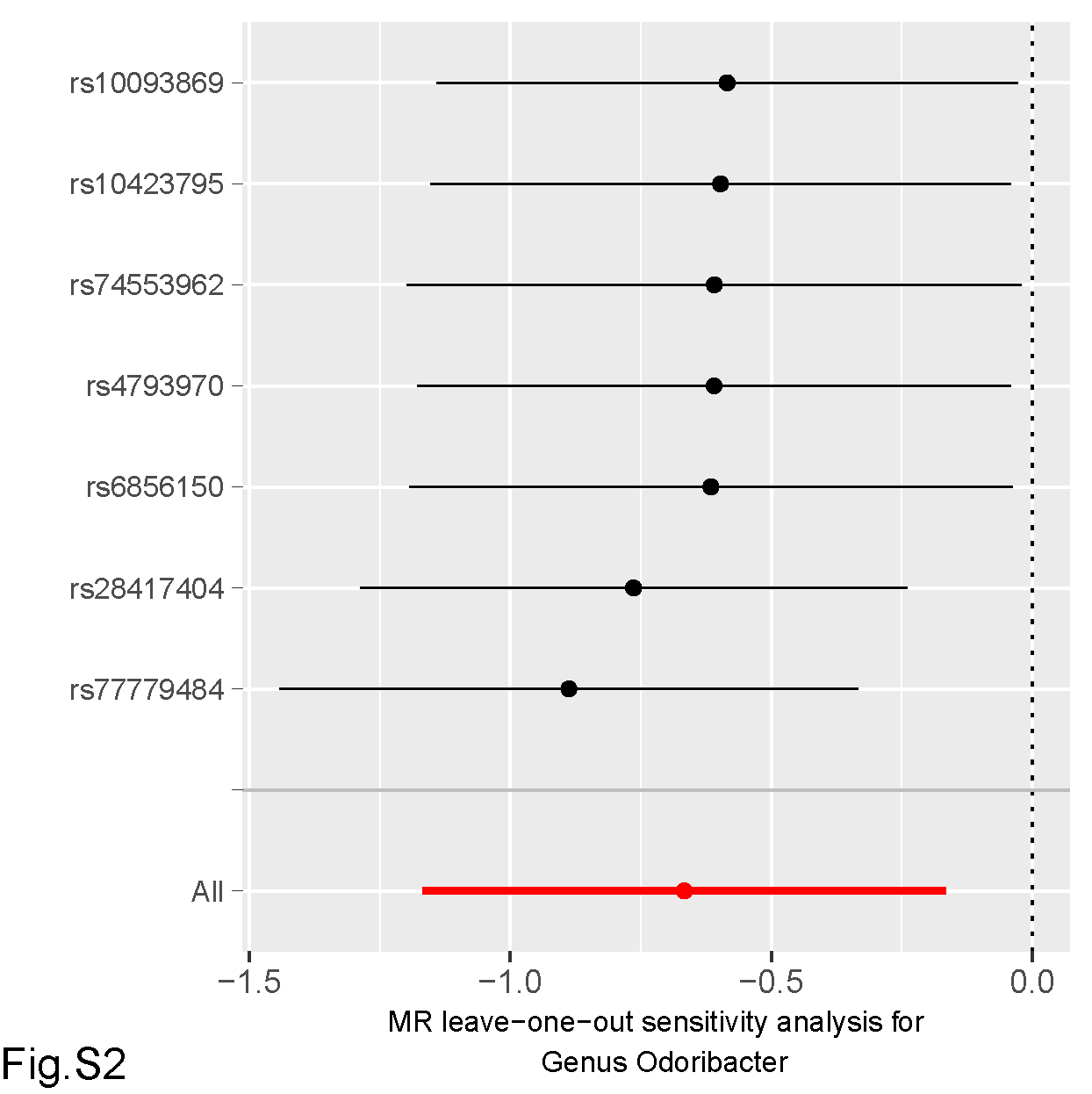

Supplement: Supplementary file 2 [file Data_Sheet_1.ZIP › supplementary_figures/Fig.S2_Genus_Odoribacter_Rplot.tif]

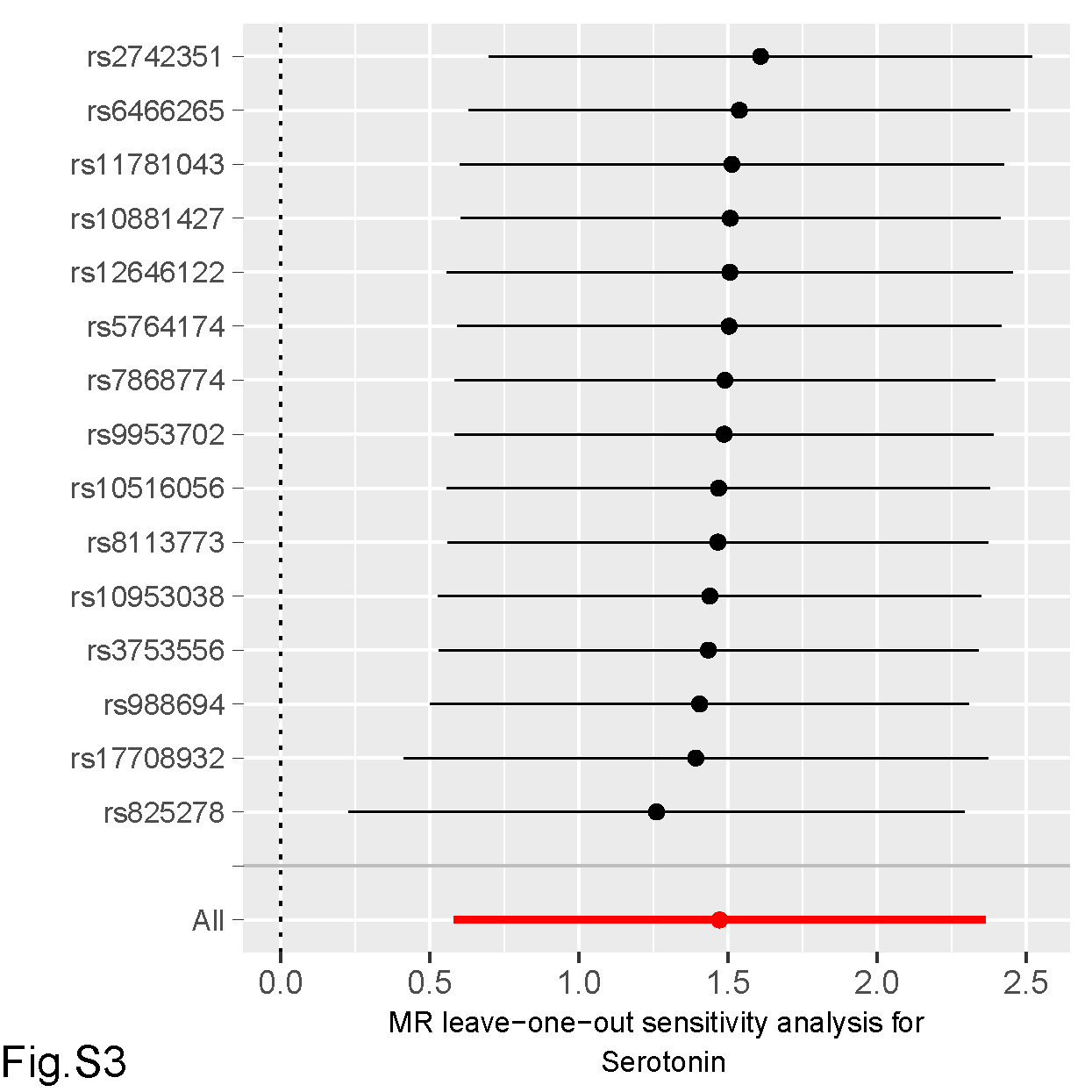

Supplement: Supplementary file 2 [file Data_Sheet_1.ZIP › supplementary_figures/Fig.S3_Serotonin _Rplot.tif]

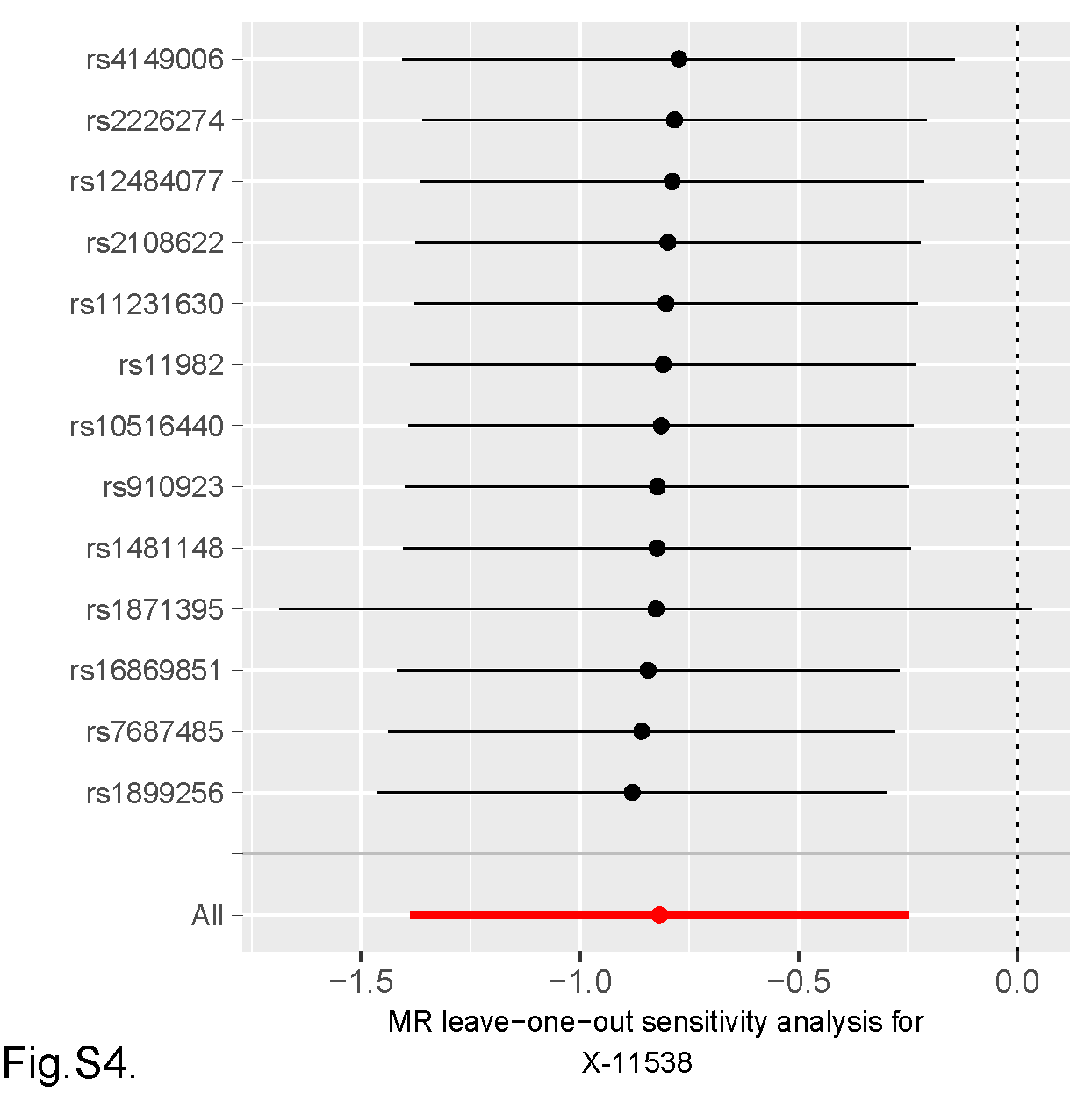

Supplement: Supplementary file 2 [file Data_Sheet_1.ZIP › supplementary_figures/Fig.S4_X-11538_Rplot.tif]

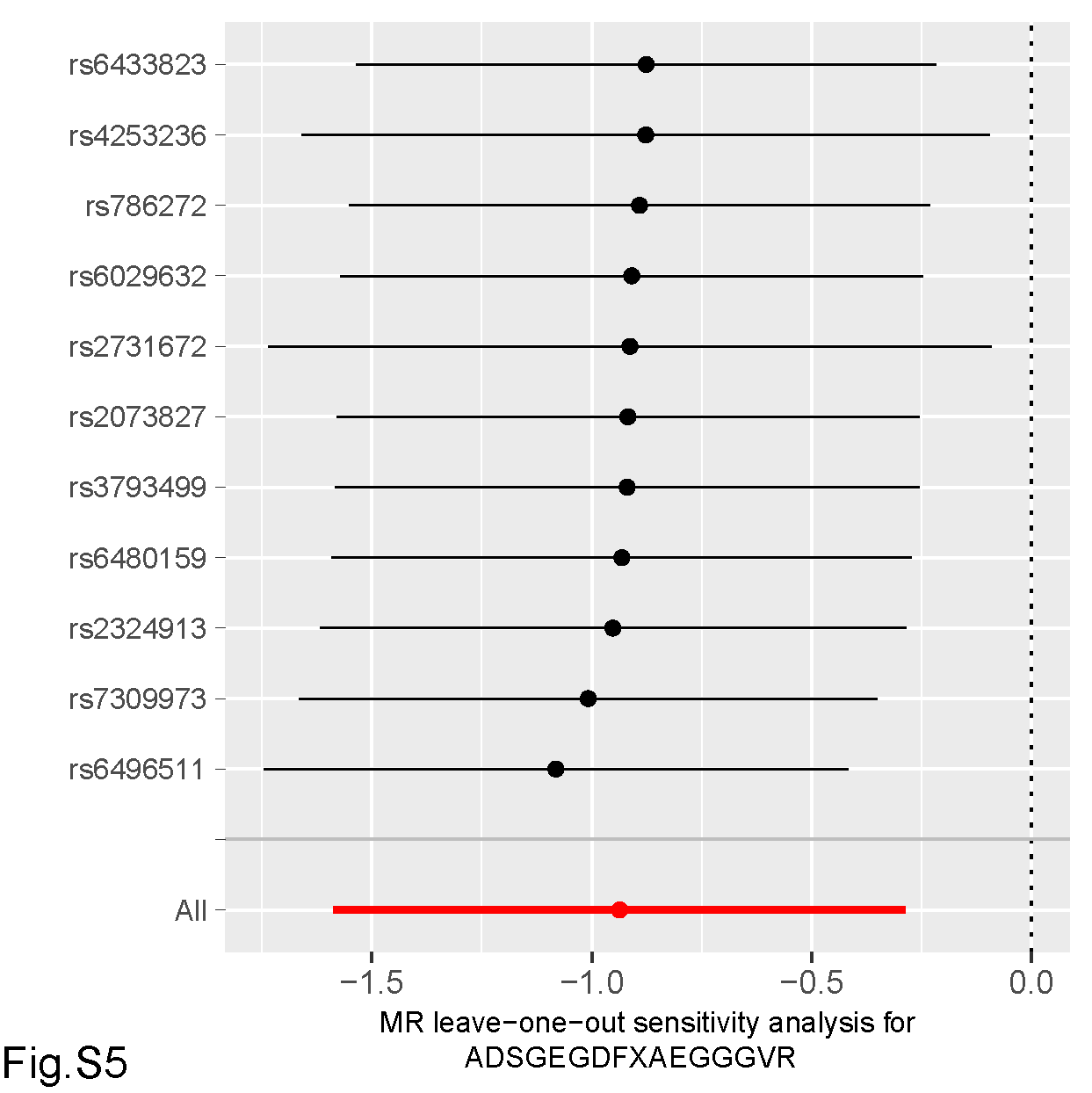

Supplement: Supplementary file 2 [file Data_Sheet_1.ZIP › supplementary_figures/Fig.S5_ADSGEGDFXAEGGGVR_Rplot.tif]

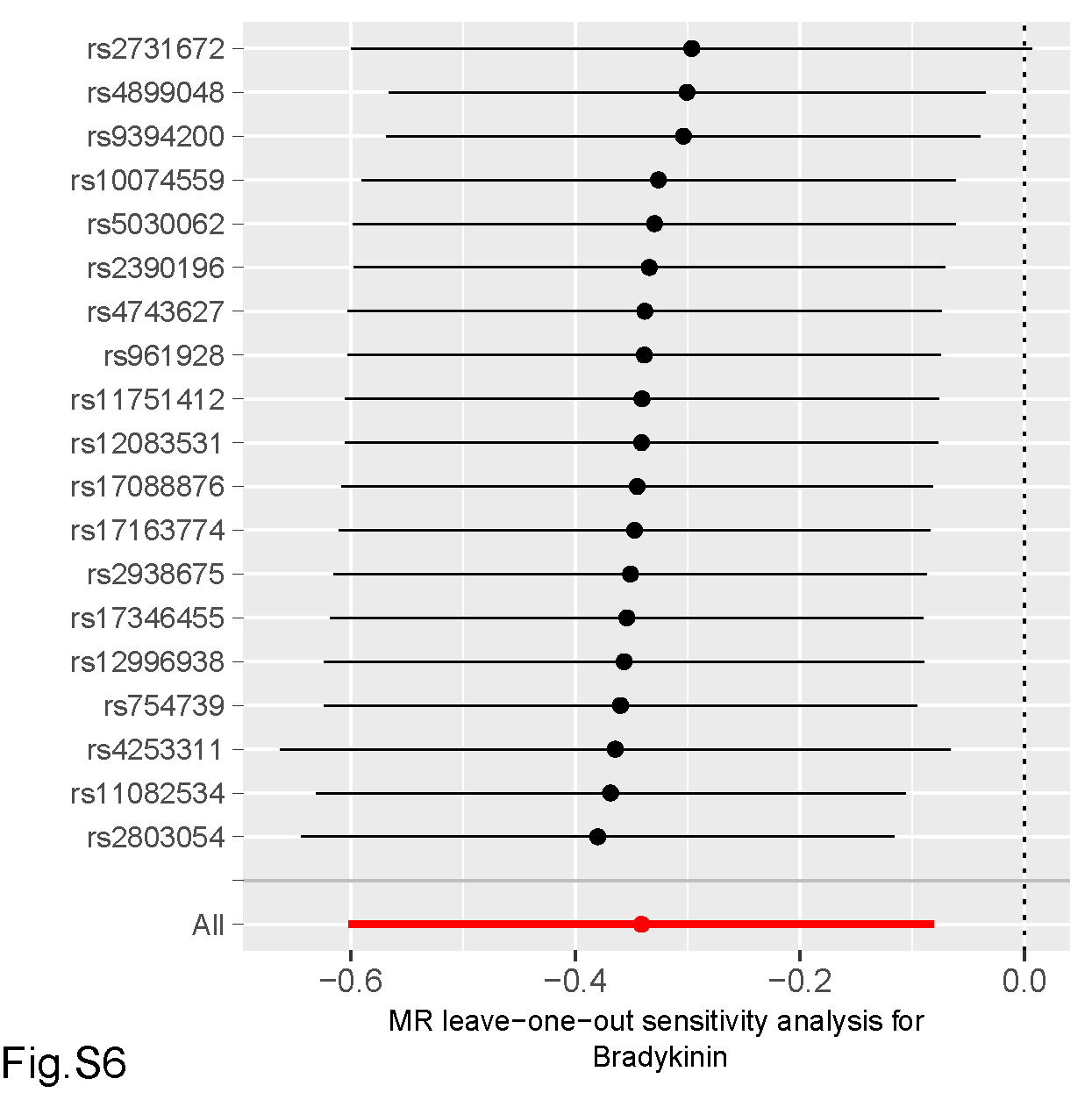

Supplement: Supplementary file 2 [file Data_Sheet_1.ZIP › supplementary_figures/Fig.S6_Bradykinin_Rplot.tif]

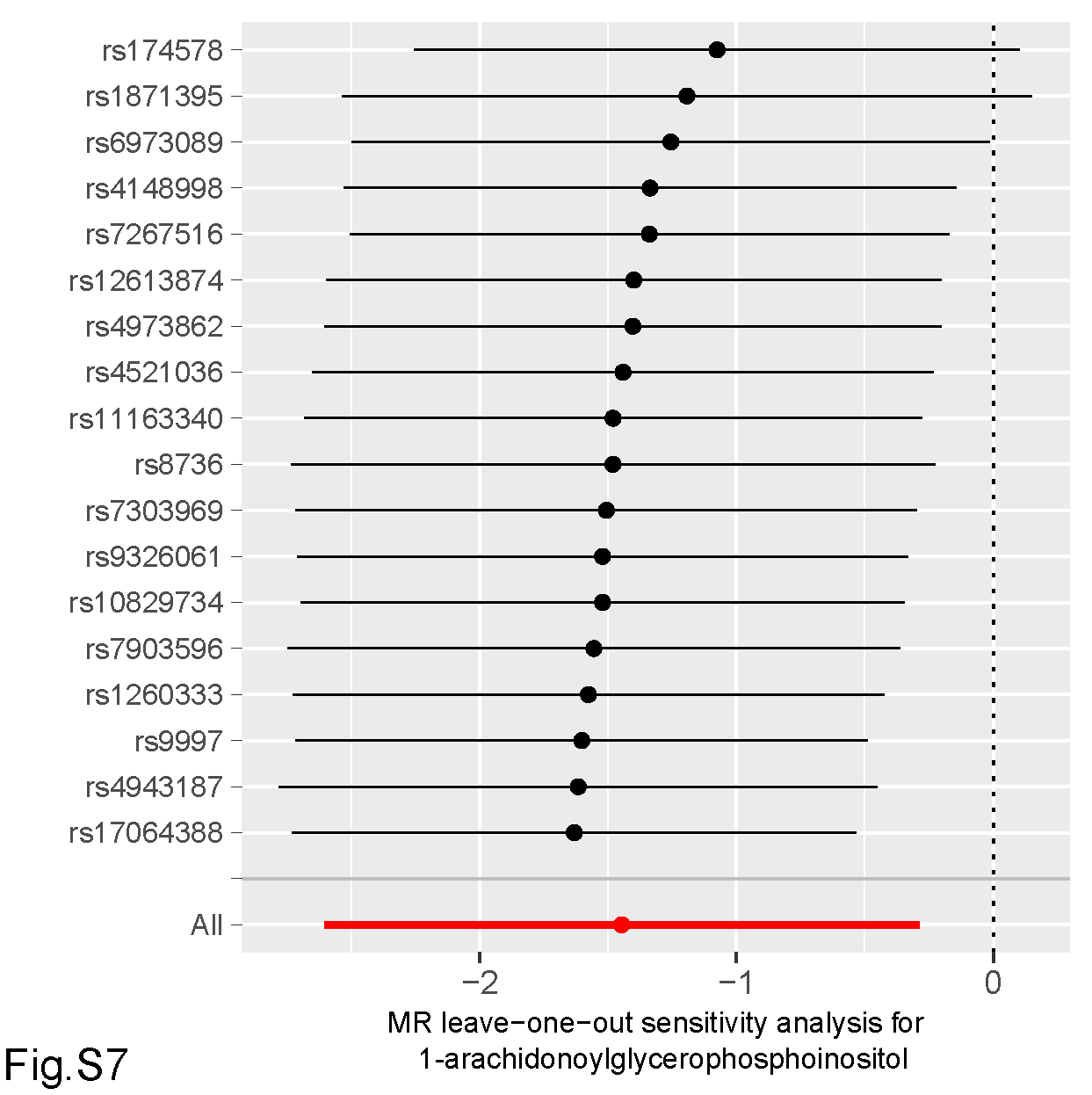

Supplement: Supplementary file 2 [file Data_Sheet_1.ZIP › supplementary_figures/Fig.S7_1-arachidonoylglycerophosphoinositol_Rplot.tif]
